# Supplementary material for: Referral to the NHS Diabetes Prevention Programme and conversion from nondiabetic hyperglycaemia to type 2 diabetes mellitus in England: A matched cohort analysis
Source: PLoS Med. 2023 Feb 27;20(2):e1004177. doi: 10.1371/journal.pmed.1004177 (PMC9970065; doi:10.1371/journal.pmed.1004177)
Supplement: S1 Appendix — (DOCX) [file pmed.1004177.s002.docx]

# Supplementary file 1 for “Independent evaluation of the effectiveness of the NHS Diabetes Prevention Programme at reducing conversion from non-diabetic hyperglycaemia to type-2 diabetes mellitus in England: a matched cohort analysis using electronic health records”

## Methodological details

### Practice Matching algorithm

Across-practice matching was used to control for potential unmeasured confounding in referrals, by matching referring practices to non-referring practices over a set time period, before matching referred patients (from the referring practice) to non-referred patients (from the matched non-referring practice). An initial step, widely used in analyses of these databases, in order to refine the selection process and reduce the population pool to relevant controls, is to use *exact matching* on certain key and complete covariates.^1-3^

We used this approach to select control (non-referred) patients, 5 to 1 if possible, to increase power, using the covariates: age, sex, general practice and date of NDH diagnosis. Age and sex are used as standard in such matching approaches, since they are complete variables that are likely to be linked to effect heterogeneity. Matching on general practice to some extent ensures similar coding practices. We added NDH diagnosis date to this preliminary step, since in early stages of this work package we identified a decreasing trend in conversion rates over time and we considered it important to ensure the distribution of cases and controls was well balanced on this covariate. Following this step, we control for all other relevant and recorded covariates in the multivariable analyses (e.g. biological parameters), with the cohort size allowing for numerous covariates to be included, without a need for propensity score matching (which is more relevant when numbers are small and the characteristics of the two populations to be compared are very different, resulting to little overlapping propensity score distributions).^4-6^

However, prior to this there is a preliminary step of matching referring and not referring practices. The steps involved there include:

#### 1 to 1 matching

1. If a practice had >1 & <20 referrals over the whole of the study period they were dropped, leaving us with practices with zero or ≥20 referrals. The decision regarding the cut offs was partly driven by the distribution.

2. Only practices which were active in the study period were used (1 April 2016 to 31 March 2020). As AURUM does not have an up-to-standard date all practices were considered active.

3. Practices with a collection date < 1 April 2018 (i.e., less than two years of data from 1 April 2016) were excluded.

4. Practices from the whole of the UK were used rather than from England alone, as control practices, to ensure enough practices were available in that pool.

5. A 1-to-1 propensity score matching approach, nearest neighbour with no replacement, was used to match between the groups. The variables included in the model were the NDH registers of each practice, for 2016, 2017, 2018 and 2019, to ensure practices of similar sizes in terms of the population of interest were matched.

6. The procedure was iterative and stratified on practice follow-up time, to ensure matching within practices of similar follow-up was prioritised.

561 pairs of practices were successfully matched, with no matches for 313 practices in the ≥20 referrals category.

#### 1 to N Matching

Steps 1 to 5 were identical. Instead of 1 to 1 nearest neighbour matching without replacement, we used nearest neighbour matching with replacement and no restrictions on the overlap assumption (ensuring all practices were matched). The procedure was not iterative as for 1 to 1 matching, but a single step where the follow-up time was included as an additional categorical covariate in the PS model. All 561 non referring and 858 referring practices were matched, with numbers of referring practices matched to one “non referring” practice ranging from 1 to 30, with a mean of 2.2 and a median of 1.

We expect the 1 to 1 practice matching approach to allow for less biased patient level matching, and hence this will form the basis of our primary analysis, with 1 to N practice matching used in sensitivity analyses.

### Defining the covariates

We extracted information on the following covariates which have previously been reported (9) to be relevant to NDH and T2DM; age, gender, BMI, HbA1c, total serum cholesterol, systolic blood pressure, diastolic blood pressure, metformin, smoking status, socioeconomic status and depression. We calculated age at referral for the matched cohort, as well as age at NDH diagnosis date. Age group was categorised into the following bands: 18-34, 35-44, 45-54, 55-64, 65-74, 75-84, and 85 years or over. The latest available measurement before the referral date, up until the previous 12 months, was used to define baseline total cholesterol, blood pressure and BMI. If such a value was not available, the measurement was set to missing. BMI values were classified into the following categories: underweight (<18.5 kg/m^2^), normal weight (18.5-24.9 kg/m^2^), overweight (25.0-29.9 kg/m^2^) and obese (>=30 kg/m^2^). Total serum cholesterol in mmol/l was categorised into: under 3.0, [3.0, 4.0), [4.0, 5.0), [5.0, 6.0] and 6.0 or over. We also quantified the multi-morbidity burden, using the Charlson Comorbidity Index (CCI), a widely used measure which assigns different weights to different conditions and includes: any malignancy, cerebrovascular disease, chronic pulmonary disease, congestive cardiac disease, dementia, HIV/AIDS, hemiplegia, lymphoproliferative disorders, metastatic solid tumour, mild liver disease, moderate and severe liver disease (CCI also includes diabetes with complications, which we necessarily excluded).^7 8^ This modified CCI was calculated using the list of validated diagnostic primary care Read codes used by Khan et al.^7^ Participants were classified as having a condition if a Read code for the condition was ever present. CCI takes integer values and was categorised as: 0, 1 to 2, 3 to 4 and greater than 4. Depression was evaluated using medical codes and therapy codes which were obtained from the code lists derived from the CPRD provided on a Cambridge University repository.^9^ For CCI we used as a condition was present as it was ever recorded in the patient’s primary care record. Participants were considered to have depression at the index date (the date of NDH diagnosis) if they were recorded as depressed either by a code or if they were on relevant medication in the last 12 months. Smoking status was determined from information based on Read codes, with information on observation and therapy files. Smoking status was recorded and categorised as “smoker”, “ex-smoker” or “never smoked” and could vary throughout the follow-up period. We used the last available information in the record, closest to the index date (referral date), and if there was no prior information on smoking status, we used the first mention of smoking status in the record following the index date, if any. Prescriptions of metformin following an NDH diagnosis before T2DM diagnosis were used to dichotomise participants as ever having received metformin following their diagnosis, or not.

### Within-practice matching details

For this analysis, we will only use data from the post-intervention period and compare NDH to T2DM conversion rates. We used only data from the post-intervention period and compare NDH to T2DM conversion rates between patients referred to the DPP versus matched patients not referred within the same practice, based on age (up to 3 years), sex and the within 180 days of NDH diagnosis.

An initial step, widely used in analyses of these databases, in order to refine the selection process and reduce the population pool to relevant controls, is to use *exact matching* on certain key and complete covariates.^1-3 10 11^We used this approach to select control (non-referred) patients, 5 to 1 if possible, to increase power, using the covariates: age, sex, general practice and date of NDH diagnosis. Age and sex are used as standard in such matching approaches, since they are complete variables that are likely to be linked to effect heterogeneity. Matching on general practice to some extent ensures similar coding practices. We added NDH diagnosis date to this preliminary step, since in early stages of this work package we identified a decreasing trend in conversion rates over time and we considered it important to ensure the distribution of those referred to NDPP and those not referred to NDPP was well balanced on this covariate. Following this step, we control for all other relevant and recorded covariates in the multivariable analyses (e.g. biological parameters), with the cohort size allowing for numerous covariates to be included, without a need for propensity score matching (which is more relevant when numbers are small and the characteristics of the two populations to be compared are very different, resulting to little overlapping propensity score distributions).^4-6^

Further analyses were carried out with relaxed NDH diagnosis date constraints (365 and 730 days) to increase the sizes of the matched samples. For each matching approach, we examined the balance in sex, age and time of NDH diagnosis between cases and a randomly selected control (to ensure a meaningful comparison). After observing good balance in all three variables for most matching approaches, we opted for an approach that maximised numbers but minimised the risk of bias due to differences in NDH diagnosis time, which we considered the biggest threat to the analyses. Thus, we decided to use the cohort matched within 3 years of age and NDH date within 365 days of diagnosis.

## Additional Results

Table A: Individuals with NDH and referral codes in CPRD (01/04/16 - 31/03/20)

|  | N | | | | | |
| --- | --- | --- | --- | --- | --- | --- |
|  | **NDH** | | **Referred** | | **Referred Declined** | |
| Date | **AURUM** | **GOLD** | **AURUM** | **GOLD** | **AURUM** | **GOLD** |
| 01/04/2016 | 354,713 | 154,369 | 9930 | 814 | 2736 | 303 |
| 01/04/2017 | 429,765 | 165,117 | 43,022 | 2835 | 16,187 | 980 |
| 01/04/2018 | 529,361 | 176,819 | 93,055 | 5597 | 39,545 | 1574 |
| 01/04/2019 | 618,967 | 188,581 | 143,388 | 7761 | 67,117 | 1961 |
| 01/04/2020 | 619,536 | 187,002 | 150,461 | 7765 | 69,847 | 1958 |

## Between practice matching

Figure A: Referred patients selected from CPRD GOLD and CPRD AURUM for Matching


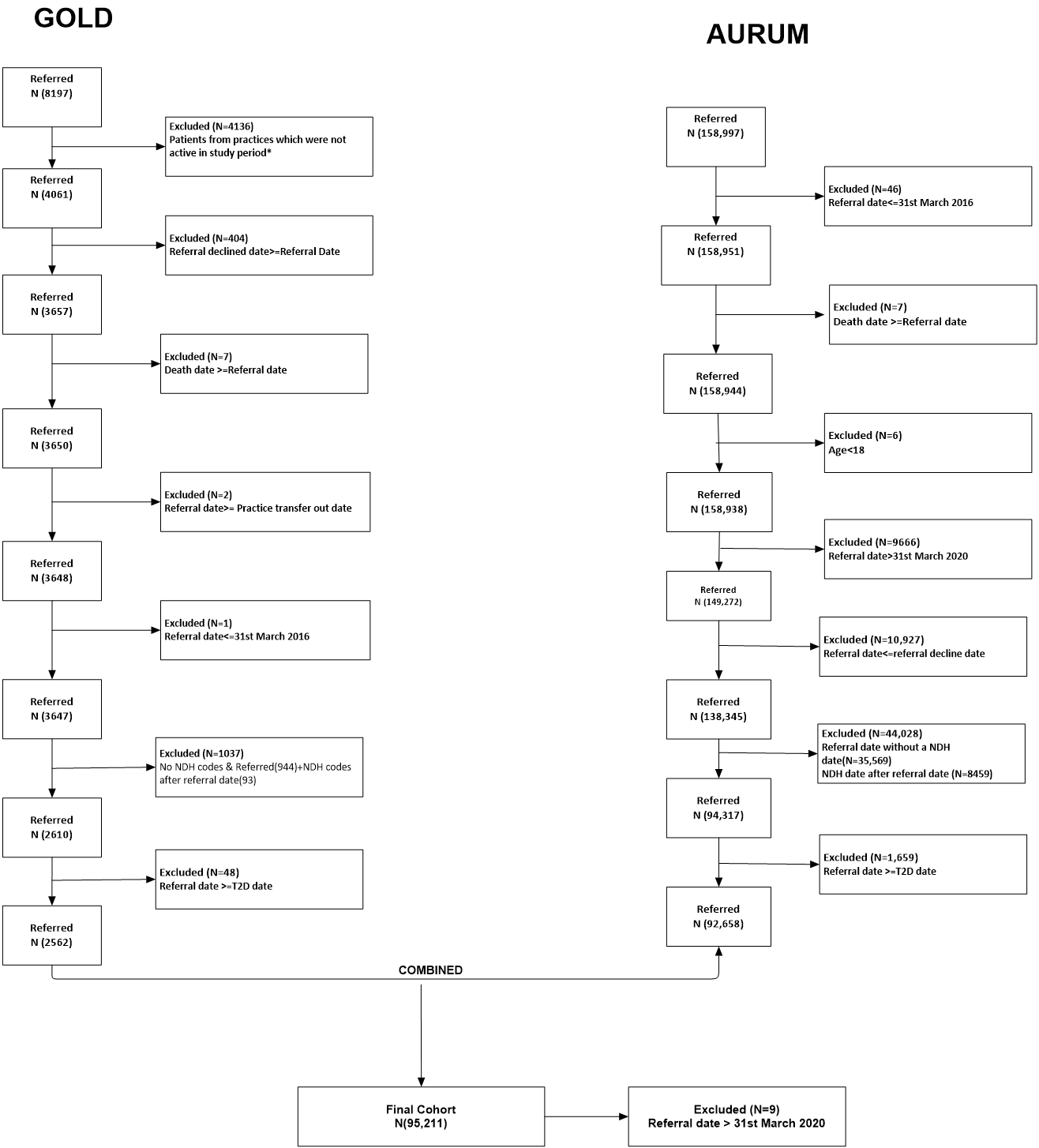


Figure B: Selection of practices for 1 to 1 Matching

Figure C: Selection of practices for 1 to N Matching

Table B: Matched cohort characteristics, mean±SD or frequency(%): one NHS DPP case matched to up to five controls, for sex, age (within 3 years) and NDH diagnosis (within 365 days).

|  | All | Referred to NDPP | Not Referred to NDPP |
| --- | --- | --- | --- |
| All | 69,801 | 18,470 | 51,331 |
| Follow up from NDH diagnosis (days) | 879.9±804.8 | 980.6±902.4) | 843.6±763.4 |
| Follow up from index date (days) | 474.9±311.3 | 482.0±317.3) | 472.4±309.1 |
| Time from NDH diagnosis to Referral date (days) | 404.9±741.7 | 498.7±835.4 | 371.2±701.9 |
| Type 2 Diabetes (T2DM) | 4432(6.3) | 1152(6.2) | 3280(6.4) |
| ≥1 year follow up or earlier T2DM conversion | 24393(62.0) | 6318 (60.4) | 18075 (62.7) |
| ≥2 years follow up or earlier T2DM conversion | 11815 (30.1) | 3229 (30.9) | 8586 (29.8) |
| ≥3 years follow up or earlier T2DM conversion | 3111 (7.9) | 919 (8.8) | 2192 (7.6) |
| Males | 33716(48.3) | 8941(48.4) | 24775(48.3) |
| Females | 36085(51.7) | 9529(51.6) | 26556(51.7) |
| Age (years)NDH diagnosis date | 62.4±11.2 | 61.9±11.6 | 62.6±11.0 |
| Age (years) Referral date | 63.6±11.3 | 63.3±11.8 | 63.7±11.1 |
| Age group (Years) NDH diagnosis date |  |  |  |
| 18-34 | 513(0.7) | 191(1.0) | 322(0.6) |
| 35-44 | 3690(5.3) | 1207(6.5) | 2483(4.8) |
| 45-54 | 12934(18.5) | 3537(19.2) | 9397(18.3) |
| 55-64 | 21601(31.0) | 5510(29.8) | 16091(31.4) |
| 65-74 | 20828(29.8) | 5319(28.8) | 15509(30.2) |
| 75-84 | 9252(13.3) | 2439(13.2) | 6813(13.3) |
| >=85 | 983(1.4) | 267(1.5) | 716(1.4) |
| BMI (kg/m2) | 31.1±6.6 | 30.8±6.4 | 31.2(6.7) |
| BMI Categories |  |  |  |
| <18.5 | 325(0.47) | 85(0.46) | 240(0.47) |
| 18.5-25 | 6328(9.1) | 1973(10.7) | 4355(8.5) |
| 25-30 | 13731(19.7) | 4300(23.3) | 9431(18.4) |
| >=30 | 21355(30.6) | 6255(33.9) | 15100(29.4) |
| Missing | 28062(40.2) | 5857(31.7) | 22205(43.3) |
| Charlson comorbidity score |  |  |  |
| None | 38371(55.0) | 10362(56.1) | 28009(54.6) |
| 1 to 2 | 19109(27.4) | 4494(24.3) | 14615(28.5) |
| 3 to 4 | 7168(10.3) | 1813(9.8) | 5355(10.4) |
| >4 | 5153(7.4) | 1801(9.8) | 3352(6.5) |
| Cholesterol (%) |  |  |  |
| <3 | 1182(1.7) | 352(1.9) | 830(1.6) |
| 3 to 4 | 8680(12.4) | 2603(14.1) | 6077(11.8) |
| 4 to 5 | 15905(22.8) | 4970(26.9) | 10935(21.3) |
| 5 to 6 | 13345(19.1) | 4441(24.0) | 8904(17.4) |
| >=6 | 8118(11.6) | 2751(14.9) | 5367(10.5) |
| Missing | 22571(32.3) | 3353(18.2) | 19218(37.4) |
| Depression | 18893(27.1) | 4275(23.2) | 14618(28.5) |
| Smoking Status |  |  |  |
| Current Smoker | 25751(36.9) | 9108(49.3) | 16643(32.4) |
| Ex-smoker | 30207(43.3) | 8029(43.5) | 22178(43.2) |
| Never smoker | 12697(18.2) | 928(5.0) | 11769(22.9) |
| Missing | 1146(1.6) | 405(2.2) | 741(1.4) |
| Metformin | 2059(3.0) | 621(3.4) | 1438(2.8) |
| Systolic Blood Pressure(mm Hg) | 135.4±14.4 | 134.9±14.1 | 135.6±14.5 |
| <120 mmHg | 6824(9.8) | 2035(11.0) | 4789(9.3) |
| {120-139}mmHg | 28387(40.7) | 8414(45.6) | 19973(38.9) |
| {140-159}mmHg | 18440(26.4) | 5075(27.5) | 13365(26.0) |
| >=160 mmHg | 2796(4.0) | 719(3.9) | 2077(4.1) |
| missing | 13354(19.1) | 2227(12.1) | 11127(21.7) |
| Diastolic Blood Pressure (mm Hg) | 79.2±9.2 | 79.4±9.1 | 79.2±9.2 |
| <80 mmHg | 28178(40.4) | 8139(44.1) | 20039(39.0) |
| {80-89}mmHg | 21457(30.7) | 6168(33.4) | 15289(29.8) |
| >=90 mmHg | 6812(9.8) | 1936(10.5) | 4876(9.5) |
| missing | 13354(19.1) | 2227(12.1) | 11127(21.7) |
| HBA1C (mmol/mol) | 43.4±3.7 | 43.5±2.3 | 43.4±3.9 |
| % with HBA1C values | 22806(32.7) | 3590(19.4) | 19216(37.4) |
| Mean days from NDH diagnosis to Referral date | 404.9±741.7 | 498.7±835.4 | 371.2±701.9 |

Table C: Matched cohort characteristics, mean±SD or frequency(%): one NHS DPP case matched to one random control, for sex, age (within 3 years) and NDH diagnosis (within 365 days).

|  | All | Referred to NDPP | Not Referred to NDPP |
| --- | --- | --- | --- |
| All | 36,940 | 18,470 | 18,470 |
| Follow up from NDH diagnosis (days) | 980.1±904.2 | 980.6±902.4 | 979.6±906.0 |
| Follow up from index date (days) | 480.4±317.4 | 482.0±317.3 | 478.8±317.6 |
| Time from NDH diagnosis to Referral date (days) | 499.7±842.0 | 498.7±835.4 | 500.7±848.6 |
| Type 2 Diabetes (T2DM) | 2524(6.8) | 1152(6.2) | 1372(7.4) |
| ≥1 year follow up or earlier T2DM conversion | 12607 (60.4) | 6318(60.4) | 6289(60.4) |
| ≥2 years follow up or earlier T2DM conversion | 6455 (30.9) | 3229(30.9) | 3226(31.0) |
| ≥3 years follow up or earlier T2DM conversion | 1815 (8.7) | 919(8.8) | 896(8.6) |
| Type 2 Diabetes | 2524(6.8) | 1152(6.2) | 1372(7.4) |
| Males | 17882(48.4) | 8941(48.4) | 8941(48.4) |
| Females | 19058(51.6) | 9529(51.6) | 9529(51.6) |
| Age (years)NDH diagnosis date | 61.9±11.6 | 61.9±11.6 | 61.9±11.6 |
| Age (years) Referral date | 63.3±11.8 | 63.3±11.8 | 63.3±11.8 |
| Age group (Years) NDH diagnosis date |  |  |  |
| 18-34 | 389(1.1) | 191(1.0) | 198(1.1) |
| 35-44 | 2348(6.4) | 1207(6.5) | 1141(6.2) |
| 45-54 | 7083(19.2) | 3537(19.2) | 3546(19.2) |
| 55-64 | 11089(30.0) | 5510(29.8) | 5579(30.2) |
| 65-74 | 10688(28.9) | 5319(28.8) | 5369(29.1) |
| 75-84 | 4804(13.0) | 2439(13.2) | 2365(12.8) |
| >=85 | 539(1.5) | 267(1.5) | 272(1.5) |
| BMI (kg/m2) | 31.1±6.6 | 30.8(6.4) | 31.5(6.9) |
| BMI Categories |  |  |  |
| <18.5 | 178(0.48) | 85(0.46) | 93(0.50) |
| 18.5-25 | 3403(9.2) | 1973(10.7) | 1430(7.7) |
| 25-30 | 7572(20.5) | 4300(23.3) | 3272(17.7) |
| >=30 | 11791(31.9) | 6255(33.9) | 5536(30.0) |
| Missing | 13996(37.9) | 5857(31.7) | 8139(44.1) |
| Charlson comorbidity score |  |  |  |
| None | 20647(55.9) | 10362(56.1) | 10285(55.7) |
| 1 to 2 | 9661(26.2) | 4494(24.3) | 5167(28.0) |
| 3 to 4 | 3669(9.9) | 1813(9.8) | 1856(10.1) |
| >4 | 2963(8.0) | 1801(9.8) | 1162(6.3) |
| Cholesterol (%) |  |  |  |
| <3 | 665(1.8) | 352(1.9) | 313(1.7) |
| 3 to 4 | 4755(12.9) | 2603(14.1) | 2152(11.7) |
| 4 to 5 | 8820(23.9) | 4970(26.9) | 3850(20.8) |
| 5 to 6 | 7614(20.6) | 4441(24.0) | 3173(17.2) |
| >=6 | 4541(12.3) | 2751(14.9) | 1790(9.7) |
| Missing | 10545(28.6) | 3353(18.2) | 7192(38.9) |
| Depression | 9535(25.8) | 4275(23.2) | 5260(28.5) |
| Smoking Status |  |  |  |
| Current Smoker | 15015(40.7) | 9108(49.3) | 5907(32.0) |
| Ex-smoker | 15879(43.0) | 8029(43.5) | 7850(42.5) |
| Never smoker | 5368(14.5) | 928(5.0) | 4440(24.0) |
| Missing | 678(1.8) | 405(2.2) | 273(1.5) |
| Metformin | 1251(3.4) | 621(3.4) | 630(3.4) |
| Systolic Blood Pressure(mm Hg) | 135.2±14.2 | 134.9±14.1 | 135.5±14.3 |
| <120 mmHg | 3722(10.1) | 2035(11.0) | 1687(9.1) |
| {120-139}mmHg | 15542(42.1) | 8414(45.6) | 7128(38.6) |
|  | **All** | **Cases** | **Controls** |
| {140-159}mmHg | 9763(26.4) | 5075(27.5) | 4688(25.4) |
| >=160 mmHg | 1435(3.9) | 719(3.9) | 716(3.9) |
| missing | 6478(17.5) | 2227(12.1) | 4251(23.0) |
| Diastolic Blood Pressure (mm Hg) | 79.3±9.1 | 79.4±9.1 | 79.1±9.2 |
| <80 mmHg | 15278(41.4) | 8139(44.1) | 7139(38.7) |
| {80-89}mmHg | 11523(31.2) | 6168(33.4) | 5355(29.0) |
| >=90 mmHg | 3661(9.9) | 1936(10.5) | 1725(9.3) |
| missing | 6478(17.5) | 2227(12.1) | 4251(23.0) |
| HBA1C (mmol/mol) | 43.5±3.8 | 43.5±2.3 | 43.5±4.4 |
| % with HBA1C values | 10324(28.0) | 3590(19.4) | 6734(36.5) |

Table D: Results summary for the intervention across all analyses

* Cox proportional hazards regression since the parametric model did not converge

|  | HR | 95% Confidence interval | |
| --- | --- | --- | --- |
| *Between practice matching, main analysis* |  |  |  |
| 1 to 1 practice matching, 1 to N patient matching with replacement, no multiple imputation | 0.80 | 0.73 | 0.87 |
| *Between practice matching, bespoke linkage sensitivity* |  |  |  |
| 1 to 1 practice matching, 1 to N patient matching with replacement, no multiple imputation | 0.76 | 0.69 | 0.84 |
| 1 to 1 practice matching, 1 to 1 patient matching with replacement, no multiple imputation | 0.75 | 0.67 | 0.84 |
| 1 to 1 practice matching, 1 to 1 patient matching with replacement, multiple imputation | 0.85 | 0.76 | 0.93 |
| 1 to 1 practice matching, 1 to 1 patient matching with replacement, multiple imputation | 0.85 | 0.75 | 0.96 |
| *Between practice matching, including no/late NDH referrals* |  |  |  |
| 1 to 1 practice matching, 1 to N patient matching with replacement, no multiple imputation | 0.77 | 0.70 | 0.84 |
| 1 to 1 practice matching, 1 to 1 patient matching with replacement, no multiple imputation | 0.78 | 0.70 | 0.88 |
| 1 to 1 practice matching, 1 to 1 patient matching with replacement, multiple imputation | 0.85 | 0.76 | 0.95 |
| 1 to 1 practice matching, 1 to 1 patient matching with replacement, multiple imputation | 0.84 | 0.74 | 0.94 |
| *Between practice matching, including region/country and database covariates* |  |  |  |
| 1 to 1 practice matching, 1 to N patient matching with replacement, no multiple imputation | 0.88 | 0.79 | 0.98 |
| 1 to 1 practice matching, 1 to 1 patient matching with replacement, no multiple imputation | 0.87 | 0.76 | 0.99 |
| 1 to 1 practice matching, 1 to 1 patient matching with replacement, multiple imputation | 0.92 | 0.82 | 1.02 |
| 1 to 1 practice matching, 1 to 1 patient matching with replacement, multiple imputation | 0.95 | 0.82 | 1.08 |
| *Between practice matching, other sensitivity analyses* |  |  |  |
| 1 to 1 practice matching, 1 to 1 patient matching with replacement, no multiple imputation | 0.78 | 0.70 | 0.87 |
| 1 to 1 practice matching, 1 to N patient matching with replacement, multiple imputation | 0.88 | 0.80 | 0.96 |
| 1 to 1 practice matching, 1 to 1 patient matching with replacement, multiple imputation | 0.90 | 0.81 | 0.99 |
| 1 to 1 practice matching, 1 to N patient matching no replacement, no multiple imputation | 0.95 | 0.86 | 1.05 |
| 1 to 1 practice matching, 1 to 1 patient matching no replacement, no multiple imputation | 0.90 | 0.80 | 1.01 |
| 1 to 1 practice matching, 1 to N patient matching no replacement, multiple imputation | 0.95 | 0.86 | 1.05 |
| 1 to 1 practice matching, 1 to 1 patient matching no replacement, multiple imputation | 0.94 | 0.83 | 1.04 |
| 1 to N practice matching, 1 to N patient matching with replacement, no multiple imputation | 0.91 | 0.85 | 0.96 |
| 1 to N practice matching, 1 to 1 patient matching with replacement, no multiple imputation | 0.89 | 0.82 | 0.95 |
| 1 to N practice matching, 1 to N patient matching with replacement, multiple imputation | 0.87 | 0.82 | 0.91 |
| 1 to N practice matching, 1 to 1 patient matching with replacement, multiple imputation | 0.87 | 0.81 | 0.93 |
| 1 to N practice matching, 1 to N patient matching no replacement, no multiple imputation | 0.99 | 0.92 | 1.07 |
| 1 to N practice matching, 1 to 1 patient matching no replacement, no multiple imputation | 0.99 | 0.91 | 1.08 |
| 1 to N practice matching, 1 to N patient matching no replacement, multiple imputation | 0.91 | 0.83 | 0.997 |
| 1 to N practice matching, 1 to 1 patient matching no replacement, multiple imputation | 0.89 | 0.81 | 0.98 |
| *Within practice matching* |  |  |  |
| 1 to N patient matching no replacement, no multiple imputation* | 1.03 | 0.99 | 1.08 |
| 1 to 1 patient matching no replacement, no multiple imputation* | 1.03 | 0.97 | 1.09 |
| 1 to N patient matching no replacement, multiple imputation | 0.85 | 0.80 | 0.91 |
| 1 to 1 patient matching no replacement, multiple imputation | 0.83 | 0.76 | 0.91 |

## Within practice matching

Figure B: Flow Chart on final Sample selection for Within Practice Matching [**Cases- Referred to NDPP**

**Controls- Not Referred to NDPP]**


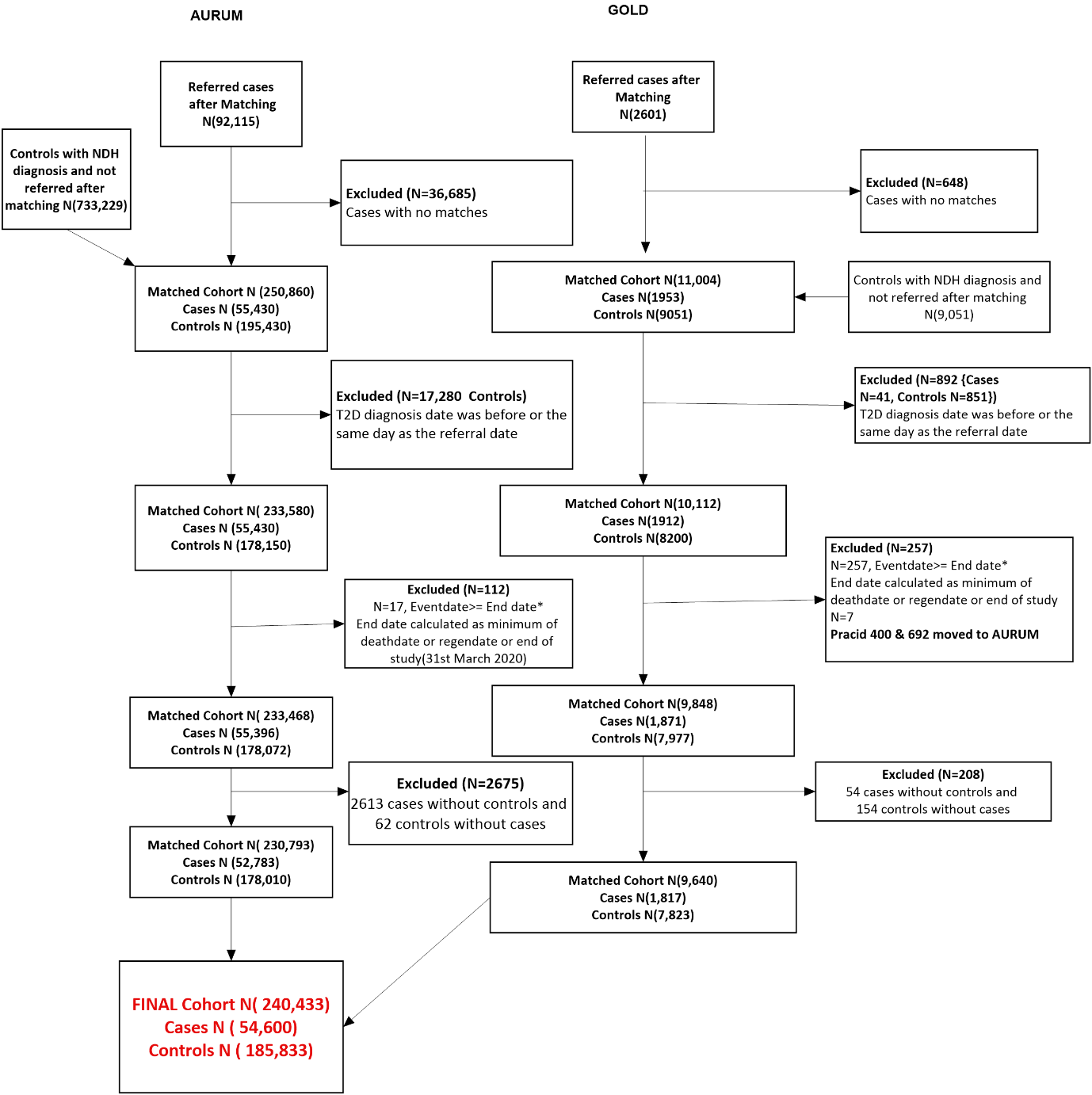


Table E: Characteristics of matched cohort {Cases are those referred to the NHS DPP and Controls are those with NDH diagnosis in primary care, matched for sex and (within 3 years) age within 365 days of NDH diagnosis date

|  | All | Referred to NDPP | Not Referred to NDPP |
| --- | --- | --- | --- |
| All | 243,397 | 57,280 | 186,117 |
| Males | 115559(47.5) | 27462(47.9) | 88097(47.3) |
| Females | 127838(52.5) | 29818(52.1) | 98020(52.7) |
| Age (years)NDH diagnosis date | 62.6±12.7 | 61.6±13.2 | 62.9±12.6 |
| Age (years) Referral date | 64.0±12.9 | 63.3±13.5 | 64.2±12.7 |
| Age group (Years) NDH diagnosis date |  |  |  |
| 18-34 | 3995(1.6) | 1299(2.3) | 2696(1.5) |
| 35-44 | 17387(7.1) | 4992(8.7) | 12395(6.7) |
| 45-54 | 44270(18.2) | 11021(19.2) | 33249(17.9) |
| 55-64 | 64921(26.7) | 15012(26.2) | 49909(26.8) |
| 65-74 | 67147(27.6) | 14728(25.7) | 52419(28.2) |
| 75-84 | 38366(15.8) | 8457(14.8) | 29909(16.1) |
| >=85 | 7311(3.0) | 1771(3.1) | 5540(3.0) |
| BMI (kg/m2) | 30.2±6.4 | 30.7±6.4 | 30(6.4) |
| BMI Categories |  |  |  |
| <18.5 | 1350(0.55) | 260(0.45) | 1090(0.59) |
| 18.5-25 | 25887(10.6) | 6369(11.1) | 19518(10.5) |
| 25-30 | 48077(19.8) | 13552(23.7) | 34525(18.6) |
| >=30 | 62801(25.8) | 19195(33.5) | 43606(23.4) |
| Missing | 105282(43.3) | 17904(31.3) | 87378(47.0) |
| Charlson comorbidity score |  |  |  |
| None | 140819(57.9) | 33967(59.3) | 106852(57.4) |
| 1 to 2 | 56487(23.2) | 13172(23.0) | 43315(23.3) |
| 3 to 4 | 25368(10.4) | 5706(10.0) | 19662(10.6) |
| >4 | 20723(8.5) | 4435(7.7) | 16288(8.8) |
| Cholesterol (%) |  |  |  |
| <3 | 4079(1.7) | 1134(2.0) | 2945(1.6) |
| 3 to 4 | 29166(12.0) | 8185(14.3) | 20981(11.3) |
| 4 to 5 | 53423(22.0) | 15386(26.9) | 38037(20.4) |
| 5 to 6 | 45637(18.8) | 13325(23.3) | 32312(17.4) |
| >=6 | 27691(11.4) | 7977(13.9) | 19714(10.6) |
| Missing | 83401(0.0) | 11273(0.0) | 72128(0.0) |
| Depression | 53520(22.0) | 13103(22.9) | 40417(21.7) |
| Smoking Status |  |  |  |
| Current Smoker | 120674(49.6) | 28336(49.5) | 92338(49.6) |
| Ex-smoker | 105445(43.3) | 24960(43.6) | 80485(43.2) |
| Never smoker | 13221(5.4) | 2991(5.2) | 10230(5.5) |
| Missing | 4057(1.7) | 993(1.7) | 3064(1.7) |
| Metformin | 8201(3.4) | 2301(4.0) | 5900(3.2) |
| Systolic Blood Pressure(mm Hg) | 134.5±14.3 | 134.5±14.2 | 134.5±14.3 |
| <120 mmHg | 25785(10.6) | 6769(11.8) | 19016(10.2) |
| {120-139}mmHg | 100185(41.2) | 26166(45.7) | 74019(39.8) |
| {140-159}mmHg | 59127(24.3) | 15199(26.5) | 43928(23.6) |
| >=160 mmHg | 8421(3.5) | 2179(3.8) | 6242(3.4) |
| missing | 49879(20.5) | 6967(12.2) | 42912(23.1) |
| Diastolic Blood Pressure (mm Hg) | 78.8±9.1 | 79.2±9.1 | 78.7±9.1 |
| <80 mmHg | 101806(41.8) | 25886(45.2) | 75920(40.8) |
| {80-89}mmHg | 70426(28.9) | 18472(32.3) | 51954(27.9) |
| {90-99} mmHg | 18156(7.5) | 5028(8.8) | 13128(7.1) |
| >=100 mmHg | 3135(1.3) | 927(1.6) | 2208(1.2) |
| missing | 49874(20.5) | 6967(12.2) | 42907(23.1) |
| HBA1C (mmol/mol) | 42.8±2.9 | 43.6±2.5 | 42.5±3.0 |
| % with HBA1C values | 32150(13.2) | 11149(19.5) | 21001(11.3) |
| Mean days from NDH diagnosis to Referral date | 545.5±854.2 | 652.1±1029.2 | 512.7±789.8 |
| Type 2 Diabetes | 14447(5.9) | 4133(7.2) | 10314(5.5) |

**References**

1. Price SJ, Stapley SA, Shephard E, et al. Is omission of free text records a possible source of data loss and bias in Clinical Practice Research Datalink studies? A case–control study. *BMJ open* 2016;6(5):e011664. doi: 10.1136/bmjopen-2016-011664

2. Nicholson BD, Aveyard P, Hobbs FDR, et al. Weight loss as a predictor of cancer and serious disease in primary care: an ISAC-approved CPRD protocol for a retrospective cohort study using routinely collected primary care data from the UK. *Diagnostic and Prognostic Research* 2018;2(1):1. doi: 10.1186/s41512-017-0019-9

3. Vinogradova Y, Coupland C, Hippisley-Cox J. Exposure to bisphosphonates and risk of gastrointestinal cancers: series of nested case-control studies with QResearch and CPRD data. *BMJ : British Medical Journal* 2013;346:f114. doi: 10.1136/bmj.f114

4. Shah BR, Laupacis A, Hux JE, et al. Propensity score methods gave similar results to traditional regression modeling in observational studies: a systematic review. *Journal of Clinical Epidemiology* 2005;58(6):550-59. doi: <https://doi.org/10.1016/j.jclinepi.2004.10.016>

5. Cepeda MS, Boston R, Farrar JT, et al. Comparison of Logistic Regression versus Propensity Score When the Number of Events Is Low and There Are Multiple Confounders. *American journal of epidemiology* 2003;158(3):280-87. doi: 10.1093/aje/kwg115

6. Zanutto EL. A Comparison of Propensity Score and Linear RegressionnAnalysis of Complex Survey Data. *Journal of Data Science* 2006;4:67-91. doi: <http://www.jds-online.com/files/JDS-233.pdf>

7. Khan NF, Perera R, Harper S, et al. Adaptation and validation of the Charlson Index for Read/OXMIS coded databases. *BMC family practice* 2010;11:1. doi: 10.1186/1471-2296-11-1 [published Online First: 2010/01/07]

8. Charlson ME, Pompei P, Ales KL, et al. A new method of classifying prognostic comorbidity in longitudinal studies: development and validation. *Journal of chronic diseases* 1987;40(5):373-83. [published Online First: 1987/01/01]

9. CPRD @ Cambridge – Codes Lists Version 1.1 October 2018 [Available from: <https://www.phpc.cam.ac.uk/pcu/cprd_cam/codelists/v11/> accessed 26/11/2018 2018.

10. Søgaard KK, Sørensen HT, Smeeth L, et al. Acute Pericarditis and Cancer Risk: A Matched Cohort Study Using Linked UK Primary and Secondary Care Data. *J Am Heart Assoc* 2018;7(16):e009428-e28. doi: 10.1161/JAHA.118.009428

11. Doyle M, While D, Mok PLH, et al. Suicide risk in primary care patients diagnosed with a personality disorder: a nested case control study. *BMC family practice* 2016;17(1):106. doi: 10.1186/s12875-016-0479-y
