# Supplementary material for: Referral to the NHS Diabetes Prevention Programme and conversion from nondiabetic hyperglycaemia to type 2 diabetes mellitus in England: A matched cohort analysis
Source: PLoS Med. 2023 Feb 27;20(2):e1004177. doi: 10.1371/journal.pmed.1004177 (PMC9970065; doi:10.1371/journal.pmed.1004177)
Supplement: S1 Analysis Plan — (DOCX) [file pmed.1004177.s003.docx]

**Overview of methods: For these analyses, we will use numerous sources of administrative data.**

First, we plan to use the National Diabetes Audit (NDA), both in itself and also linked at the individual patient level to the DPP Minimum Dataset (MDS). The NDA plus MDS will provide a national-level source of data on patients with NDH, including their referral and participation in the DPP, and subsequent outcomes including diabetes diagnosis, HcA1c and BMI, across all GP practices. Initially data from the National Diabetes Audit was not considered for DIPLOMA as it lacked data on patients with non-diabetic hyperglycaemia, thus not allowing conversion to diabetes to be evaluated. That information has now been added (including retrospectively). Providing that the NDA dataset satisfies various completeness and quality checks, the NDA+MDS will be used as the primary data source.

The second major source of routine data will be the Clinical Practice Research Datalink (CPRD) GOLD, a large database of administrative primary care data. CPRD covers fewer GP practices than the NDA+MDS, though is still substantial in itself, and will become the primary data source should NDA+MDS be found not suitable. The database has been active since the 1980s with high quality data becoming available after 2000 with the introduction of the Quality and Outcomes Framework (QOF).^1^ Complete data on all aspects of care (diagnoses, referrals, treatments, tests) have been collected from over 500 practices each financial year, covering approximately 7% of the UK population.^2^ The CPRD GOLD population is generally representative of the UK population, especially in terms of practice and patient deprivation, although it is largely tied to a single clinical computer system (Vision),^3^ and as a consequence the North-East of England is somewhat under-represented.^4^ The CPRD data can be linked to Hospital Episode Statistics (HES) and Office for National Statistics (ONS) data, allowing the construction of a more complete patient journey through primary and secondary care and the mapping of causes of mortality (e.g. diabetes, cardiovascular). From October 2017, CPRD has also access to GP practices with the IT software system EMIS Web, which is used in 56% of English practices. This larger data resource, which ensures better population coverage has similar characteristics to the GOLD database described above.

A third data source will be the general practice diabetes registers as collected for the QoF,^5^ and related information about the general practices themselves. We will make use of free public health datasets from the ONS and the Health & Social Care Information Centre (HSCIC), including geographical data, 2011 census based population estimates, deprivation and rurality information,^6^ to map diabetes at a low population level and also to scale up our findings to a national level. We will also obtain point of interest data from the Ordnance Survey (OS).^7^ All these datasets will be linked at a low population level and will be used to map diabetes and non-diabetic hyperglycaemia prevalence rates and their associations with area and population characteristics nationally. Non-diabetic hyperglycaemia data in practice registers should become available after implementation of the NHS DPP nationally, and will be linked with existing datasets.

**Research question WP 5.2 - What is the effectiveness of the NHS DPP at reducing the conversion of non-diabetic hyperglycaemia to diabetes?**

The primary objective of the NHS DPP is to reduce, or at least slow, the rate at which patients with non-diabetic hyperglycaemia go on to develop a full diabetes diagnosis. In line with this, the main outcome in these analyses will be the conversion of non-diabetic hyperglycaemia to diabetes.

**Methods**: The primary analysis for assessing the effectiveness of the NHS DPP will use the NDA+MDS if assessed as fit for that purpose, otherwise the work will be done in the CPRD. For robustness, we will address the question using two different research designs.

**WP 5.2 design 1**: We will use all practices participating in the NHS DPP, and compare the conversion rate from non-diabetic hyperglycaemia to diabetes in patients prior to the start of the NHS DPP to the rate afterwards in equivalent patients. To accomplish this, we will match pre-intervention cases of non-diabetic hyperglycaemia^8^ to post-intervention cases at the same practice and with the same practitioner if possible (excluding cases with a previous diabetes diagnoses). We will then compare the rates of conversion to diabetes within 2 years between these groups.

Earlier, we confirmed that risk-of-diabetes Read codes are already routinely being used. As the NHS DPP-specific Read codes are not available for patients prior to the scheme, we will conduct an initial exercise to identify the group of pre-existing codes that best identify patients classed as eligible using the NHS DPP-specific codes. We will then use the identified codes to classify patients as scheme- eligible both pre- and post-NHS DPP, to ensure comparability. We will use propensity score methods for the matching. In the logistic regression model to calculate the score we will include: age, sex, region, deprivation, QOF comorbidities and all available biological parameters (e.g. body mass index and HbA1c levels at baseline). Data will be complete for all covariates except the biological parameters, for which we expect a very high level of completeness for this group of patients.

Nevertheless, we will use appropriate multiple imputation methods for longitudinal data if needed.^9^

We will compare the conversion rates to diabetes between the two matched groups. Since practices are unlikely to refer all their cases of non-diabetic hyperglycaemia to the scheme (because of capacity limits), we will also compare those patients who were referred to their matched pre-intervention controls, while recognising that this comparison will be subject to confounding with any selection bias.

For the analyses we will use both logistic regression models to compare 2-year conversion rates (and over longer time-periods if the data are available) and more appropriate Cox proportional hazards and competing-risks survival regression models to account for censoring and competing risks (e.g. deaths). We will include a region covariate in the models to assess the heterogeneity of the effect, i.e. to investigate if the observed effect varies greatly across regions.

Sample size: We estimate 26,581 participants (people receiving at least one DPP treatment session) will be needed nationally to achieve 90% power to detect an intention to treat risk reduction of 25%. The NHS DPP aims to provide places for 100,000 patients each year by 2020. We assume a balanced design, alpha level of 5%, a conservative baseline 2-year conversion rate to diabetes of 7.5%,^10^ and an intention to treat risk reduction of 25% in DPP participants (i.e. 2-year conversion rate of 5.625% or OR=0.735).^11^ On this basis, we would need a total of 7331 patients to achieve 90% power to detect that level of risk reduction. As demonstrated earlier, we will have many times that number for the pre-intervention group (71,521 cases associated with non-diabetic hyperglycaemia in financial year 2015-16). For the post intervention group we would need 3,666 patients, a figure we would expect to be available in the NDA+MDS (and also CPRD if 52,371 invitations to the scheme are administered nationally; since the CPRD covers approximately 7% of the UK population). Assuming a 5 (pre- intervention) to 1 (NHS DPP) design and with all other assumptions unchanged, we would need a total of 11,164 to achieve 90% power, or 1,861 intervention patients. Only 26,581 participants nationally would be required to identify this number of participants in the CPRD.

We will explore a number of secondary outcomes. For hospitalisation, we will use all cases of hospitalisation and cases where the main reason for hospitalisation was diabetes, within 2 years of the index date (referral to the scheme). For primary care visits, we will use all visits to primary care within 2 years of the index date. For biological parameters (HbA1c and BMI) we will use the last available measurement within 1 year and 2 years of the index date (since HbA1c and BMI are expected to respond within a shorter period). We have developed relevant methodologies for BMI prediction and have used other methodological tools for multiple imputation of missing data, which we will consider using in this context.

All analyses will be repeated for the secondary outcomes with small changes in the analytic models: Poisson regressions for hospitalisations and primary care visits; linear regressions for HbA1c levels and weight/BMI; Cox proportional hazards regression for deaths. Sensitivity analyses will be used to assess the robustness of the results to different assumptions about patient eligibility, choice of co- variates, and modelling options (e.g. multivariable regression instead of matching).

**WP 5.2 design 2**: For this analysis, we will only use data from the post-intervention period and compare non-diabetic hyperglycaemia-to-diabetes conversion rates. Our main analyses will involve within practice matching, of patients referred to the scheme versus matched patients not referred. However, we will also use across practice matching to control for potential unmeasured confounding in referrals, by matching referring practices to non-referring practices over a set time period, before matching referred patients (from the referring practice) to non-referred patients (from the matched non-referring practice). In the within practice matching we will match patients on age, sex, time of NDH diagnosis and practice. We will attempt to include more controls to increase power (5 to 1, if possible). In the across practice matching we will match referring to non-referring practices within each English region, and patients will be matched on age, sex and time of NDH diagnosis.

Analyses will closely resemble those in the first design for both the primary (conversion to diabetes) and secondary outcomes (hospitalisation, primary care visits, HbA1c and BMI/weight levels, and death), and we will also investigate effect heterogeneity across regions in this design as well. The power considerations for an intention to treat analysis (comparing not invited and invited) are the same as in the first design. The analyses are potentially subject to confounding due to selection bias (at the patient and practice level, respectively), but no more than other types of comparisons based on CPRD or routinely collected health records. There are a variety of methods available for assessing/adjusting for unmeasured confounding,^12-15^ and we will apply selected methods to determine the likelihood that any results could be accounted for by this.

**Data linkage**

Within the dataset we will have access to information on personal characteristics, diagnoses of non- diabetic hyperglycaemia (NDH - and its predecessors impaired glucose regulation – IGR - and impaired glucose tolerance - IGT), exposure to the NHS-DPP and the primary outcome (progression to diabetes). There will be no need for us to link patient-level variables from different data sources. CRPD data has already been successfully linked to Hospital Episode Statistics (HES) and Office for National Statistics (ONS) data, allowing the construction of a more complete patient journey through primary and secondary care and the mapping of causes of mortality (e.g. diabetes, cardiovascular). To complement the patient level analyses and deliver a more complete evaluation of the scheme, we will also use various national databases of aggregate data such as NDA at the practice or low geographical level. The aggregate datasets will be linked at the practice level using the NHS practice ID, and then all relevant data will be linked using the NHS attribution dataset (which links primary care patients to ONS lower super output areas using their residence postcode) to low-level geographical areas where they will be combined with deprivation and relevant census covariates. The patient-level data and the aggregate data databases will not be linked.

**References**

1. Olier I, Springate DA, Ashcroft DM, et al. Modelling Conditions and Health Care Processes in Electronic Health Records: An Application to Severe Mental Illness with the Clinical Practice Research Datalink. *PLoS One* 2016;11(2):e0146715. doi: 10.1371/journal.pone.0146715 [published Online First: 2016/02/27]

2. Herrett E, Gallagher AM, Bhaskaran K, et al. Data Resource Profile: Clinical Practice Research Datalink (CPRD). *International journal of epidemiology* 2015;44(3):827-36. doi: 10.1093/ije/dyv098 [published Online First: 2015/06/08]

3. Kontopantelis E, Buchan I, Reeves D, et al. Relationship between quality of care and choice of clinical computing system: retrospective analysis of family practice performance under the UK's quality and outcomes framework. *BMJ open* 2013;3(8):e003190. doi: 10.1136/bmjopen-2013-003190

4. Kontopantelis E, Springate D, Reeves D, et al. Withdrawing performance indicators: retrospective analysis of general practice performance under UK Quality and Outcomes Framework. *BMJ : British Medical Journal* 2014;348:g330. doi: 10.1136/bmj.g330

5. Roland M, Guthrie B. Quality and Outcomes Framework: what have we learnt? *BMJ* 2016;354 doi: 10.1136/bmj.i4060

6. Government CaL. The English Indices of Deprivation 2010: Department for Communities and Local Government, 2010.

7. Survey O. Points of Interest 2016 [Available from: <https://www.ordnancesurvey.co.uk/business-government/products/points-of-interest>.

8. Mainous AG, 3rd, Tanner RJ, Baker R, et al. Prevalence of prediabetes in England from 2003 to 2011: population-based, cross-sectional study. *BMJ open* 2014;4(6):e005002. doi: 10.1136/bmjopen-2014-005002 [published Online First: 2014/06/11]

9. Welch C, Bartlett J, Petersen I. Application of multiple imputation using the two-fold fully conditional specification algorithm in longitudinal clinical data. *The Stata journal* 2014;14(2):418-31. [published Online First: 2014/11/25]

10. Tabák AG, Herder C, Rathmann W, et al. Prediabetes: A high-risk state for developing diabetes. *Lancet* 2012;379(9833):2279-90. doi: 10.1016/S0140-6736(12)60283-9

11. Nuzhat B Ashra, Rebecca Spong, Patrice Carter, et al. A systematic review and metaanalysis assessing the effectiveness of pragmatic lifestyle interventions for the prevention of type 2 diabetes mellitus in routine practice. In: England PH, ed., 2015.

12. Pressler TR, Kaizar EE. The use of propensity scores and observational data to estimate randomized controlled trial generalizability bias. *Stat Med* 2013;32(20):3552-68. doi: 10.1002/sim.5802 [published Online First: 04/01]

13. Danaei G, Rodríguez LA, Cantero OF, et al. Observational data for comparative effectiveness research: an emulation of randomised trials of statins and primary prevention of coronary heart disease. *Statistical methods in medical research* 2013;22(1):70-96. doi: 10.1177/0962280211403603 [published Online First: 2011/10/22]

14. Groenwold RHH, Nelson DB, Nichol KL, et al. Sensitivity analyses to estimate the potential impact of unmeasured confounding in causal research. *International journal of epidemiology* 2009;39(1):107-17. doi: 10.1093/ije/dyp332

15. Stuart EA, Cole SR, Bradshaw CP, et al. The use of propensity scores to assess the generalizability of results from randomized trials. *J R Stat Soc Ser A Stat Soc* 2001;174(2):369-86. doi: 10.1111/j.1467-985X.2010.00673.x
