# Supplementary material for: Referral to the NHS Diabetes Prevention Programme and conversion from nondiabetic hyperglycaemia to type 2 diabetes mellitus in England: A matched cohort analysis
Source: PLoS Med. 2023 Feb 27;20(2):e1004177. doi: 10.1371/journal.pmed.1004177 (PMC9970065; doi:10.1371/journal.pmed.1004177)
Supplement: S1 Codelist — (DOCX) [file pmed.1004177.s004.docx]

**Supplementary file 3 - Codelist**

**Table 1: Read codes used to diagnose Type 2 Diabetes Mellitus**

| **Medcode** | **Readcode** | **Description** |
| --- | --- | --- |
| 506 | C100112 | Non-insulin dependent diabetes mellitus |
| 758 | C10F.00 | Type 2 diabetes mellitus |
| 1407 | C10FJ00 | Insulin treated Type 2 diabetes mellitus |
| 4513 | C109.00 | Non-insulin dependent diabetes mellitus |
| 5884 | C109.11 | NIDDM - Non-insulin dependent diabetes mellitus |
| 8403 | C109700 | Non-insulin dependent diabetes mellitus - poor control |
| 12640 | C10FC00 | Type 2 diabetes mellitus with nephropathy |
| 12736 | C10F500 | Type 2 diabetes mellitus with gangrene |
| 17262 | C109600 | Non-insulin-dependent diabetes mellitus with retinopathy |
| 17859 | C109.12 | Type 2 diabetes mellitus |
| 18143 | C109G11 | Type II diabetes mellitus with arthropathy |
| 18209 | C109012 | Type 2 diabetes mellitus with renal complications |
| 18219 | C109.13 | Type II diabetes mellitus |
| 18264 | C109J12 | Insulin treated Type II diabetes mellitus |
| 18278 | C109J00 | Insulin treated Type 2 diabetes mellitus |
| 18390 | C10FM00 | Type 2 diabetes mellitus with persistent microalbuminuria |
| 18425 | C10FB00 | Type 2 diabetes mellitus with polyneuropathy |
| 18496 | C10F600 | Type 2 diabetes mellitus with retinopathy |
| 18777 | C10F000 | Type 2 diabetes mellitus with renal complications |
| 22884 | C10F.11 | Type II diabetes mellitus |
| 24458 | C109711 | Type II diabetes mellitus - poor control |
| 24693 | C109G00 | Non-insulin dependent diabetes mellitus with arthropathy |
| 24836 | C109C12 | Type 2 diabetes mellitus with nephropathy |
| 25041 | ZC2CA00 | Dietary advice for type II diabetes |
| 25591 | C10FQ00 | Type 2 diabetes mellitus with exudative maculopathy |
| 25627 | C10F700 | Type 2 diabetes mellitus - poor control |
| 26054 | C10FL00 | Type 2 diabetes mellitus with persistent proteinuria |
| 29979 | C109900 | Non-insulin-dependent diabetes mellitus without complication |
| 32627 | C10FN00 | Type 2 diabetes mellitus with ketoacidosis |
| 34268 | C10F200 | Type 2 diabetes mellitus with neurological complications |
| 34450 | C10FK00 | Hyperosmolar non-ketotic state in type 2 diabetes mellitus |
| 34912 | C109400 | Non-insulin dependent diabetes mellitus with ulcer |
| 35385 | C10FH00 | Type 2 diabetes mellitus with neuropathic arthropathy |
| 36633 | C109K00 | Hyperosmolar non-ketotic state in type 2 diabetes mellitus |
| 36695 | C10D.00 | Diabetes mellitus autosomal dominant type 2 |
| 37648 | C109J11 | Insulin treated non-insulin dependent diabetes mellitus |
| 37806 | C10FF00 | Type 2 diabetes mellitus with peripheral angiopathy |
| 40401 | C109500 | Non-insulin dependent diabetes mellitus with gangrene |
| 42762 | C109612 | Type 2 diabetes mellitus with retinopathy |
| 43227 | C10F311 | Type II diabetes mellitus with multiple complications |
| 43785 | C109D00 | Non-insulin dependent diabetes mellitus with hypoglyca coma |
| 44779 | C109E12 | Type 2 diabetes mellitus with diabetic cataract |
| 44982 | C10FE00 | Type 2 diabetes mellitus with diabetic cataract |
| 45467 | C109B00 | Non-insulin dependent diabetes mellitus with polyneuropathy |
| 45913 | C109712 | Type 2 diabetes mellitus - poor control |
| 45919 | C109212 | Type 2 diabetes mellitus with neurological complications |
| 46150 | C109512 | Type 2 diabetes mellitus with gangrene |
| **Medcode** | **Readcode** | **Description** |
| 46917 | C10FD00 | Type 2 diabetes mellitus with hypoglycaemic coma |
| 47315 | C10F711 | Type II diabetes mellitus - poor control |
| 47321 | C10F100 | Type 2 diabetes mellitus with ophthalmic complications |
| 47409 | C109B11 | Type II diabetes mellitus with polyneuropathy |
| 47816 | C109H11 | Type II diabetes mellitus with neuropathic arthropathy |
| 47954 | C10F900 | Type 2 diabetes mellitus without complication |
| 48192 | C109E11 | Type II diabetes mellitus with diabetic cataract |
| 49074 | C10F400 | Type 2 diabetes mellitus with ulcer |
| 49655 | C10F611 | Type II diabetes mellitus with retinopathy |
| 49869 | C109G12 | Type 2 diabetes mellitus with arthropathy |
| 50225 | C109011 | Type II diabetes mellitus with renal complications |
| 50429 | C109100 | Non-insulin-dependent diabetes mellitus with ophthalm comps |
| 50527 | C10FB11 | Type II diabetes mellitus with polyneuropathy |
| 50609 | L180600 | Pre-existing diabetes mellitus, non-insulin-dependent |
| 50813 | C109A11 | Type II diabetes mellitus with mononeuropathy |
| 51756 | C10FP00 | Type 2 diabetes mellitus with ketoacidotic coma |
| 52303 | C109000 | Non-insulin-dependent diabetes mellitus with renal comps |
| 53392 | C10F911 | Type II diabetes mellitus without complication |
| 54899 | C109F11 | Type II diabetes mellitus with peripheral angiopathy |
| 55075 | C109411 | Type II diabetes mellitus with ulcer |
| 55842 | C109200 | Non-insulin-dependent diabetes mellitus with neuro comps |
| 56268 | C109D11 | Type II diabetes mellitus with hypoglycaemic coma |
| 56803 | C107400 | NIDDM with peripheral circulatory disorder |
| 57278 | C10F011 | Type II diabetes mellitus with renal complications |
| 58604 | C109611 | Type II diabetes mellitus with retinopathy |
| 59253 | C10FG00 | Type 2 diabetes mellitus with arthropathy |
| 59365 | C109C00 | Non-insulin dependent diabetes mellitus with nephropathy |
| 59725 | C109111 | Type II diabetes mellitus with ophthalmic complications |
| 60699 | C109F12 | Type 2 diabetes mellitus with peripheral angiopathy |
| 60796 | C10FL11 | Type II diabetes mellitus with persistent proteinuria |
| 61071 | C109D12 | Type 2 diabetes mellitus with hypoglycaemic coma |
| 62107 | C109511 | Type II diabetes mellitus with gangrene |
| 62146 | C109300 | Non-insulin-dependent diabetes mellitus with multiple comps |
| 62674 | C10FA00 | Type 2 diabetes mellitus with mononeuropathy |
| 63690 | C10FR00 | Type 2 diabetes mellitus with gastroparesis |
| 64571 | C109C11 | Type II diabetes mellitus with nephropathy |
| 64668 | C10FJ11 | Insulin treated Type II diabetes mellitus |
| 65267 | C10F300 | Type 2 diabetes mellitus with multiple complications |
| 65704 | C109412 | Type 2 diabetes mellitus with ulcer |
| 66965 | C109H12 | Type 2 diabetes mellitus with neuropathic arthropathy |
| 67905 | C109211 | Type II diabetes mellitus with neurological complications |
| 69278 | C109E00 | Non-insulin depend diabetes mellitus with diabetic cataract |
| 70316 | C109112 | Type 2 diabetes mellitus with ophthalmic complications |
| 72320 | C109A00 | Non-insulin dependent diabetes mellitus with mononeuropathy |
| 83532 | 66Ao.00 | Diabetes type 2 review |
| 85991 | C10FM11 | Type II diabetes mellitus with persistent microalbuminuria |
| 91646 | C10F411 | Type II diabetes mellitus with ulcer |
| 93727 | C10FE11 | Type II diabetes mellitus with diabetic cataract |
| 95351 | C10FA11 | Type II diabetes mellitus with mononeuropathy |
| **Medcode** | **Readcode** | **Description** |
| 98616 | C10F211 | Type II diabetes mellitus with neurological complications |
| 98723 | C10FD11 | Type II diabetes mellitus with hypoglycaemic coma |
| 100964 | C10F111 | Type II diabetes mellitus with ophthalmic complications |
| 101801 | 66At100 | Type II diabetic dietary review |
| 102201 | C10FC11 | Type II diabetes mellitus with nephropathy |
| 102611 | 66At111 | Type 2 diabetic dietary review |
| 103902 | C10FG11 | Type II diabetes mellitus with arthropathy |
| 104323 | C10F511 | Type II diabetes mellitus with gangrene |
| 104639 | C10FF11 | Type II diabetes mellitus with peripheral angiopathy |
| 105784 | C109912 | Type 2 diabetes mellitus without complication |
| 106061 | C10FP11 | Type II diabetes mellitus with ketoacidotic coma |
| 106528 | C10FN11 | Type II diabetes mellitus with ketoacidosis |
| 107701 | C10FK11 | Hyperosmolar non-ketotic state in type II diabetes mellitus |
| 108005 | C109312 | Type 2 diabetes mellitus with multiple complications |
| 109103 | C109911 | Type II diabetes mellitus without complication |
| 109197 | C10FH11 | Type II diabetes mellitus with neuropathic arthropathy |
| 109865 | C109B12 | Type 2 diabetes mellitus with polyneuropathy |
| 111798 | C10FQ11 | Type II diabetes mellitus with exudative maculopathy |
