# Supplementary material for: Referral to the NHS Diabetes Prevention Programme and conversion from nondiabetic hyperglycaemia to type 2 diabetes mellitus in England: A matched cohort analysis
Source: PLoS Med. 2023 Feb 27;20(2):e1004177. doi: 10.1371/journal.pmed.1004177 (PMC9970065; doi:10.1371/journal.pmed.1004177)
Supplement: Attachment — Submitted filename: Response_Final.docx [file pmed.1004177.s005.docx]

-----------------------------------------------------------
Requests from the editors:

1)      Please include line numbers in the next draft.

***Line numbers have now been added***

2)      Please revise your title according to PLOS Medicine's style. Your title must be nondeclarative and not a question. It should begin with main concept if possible. Please place the study design only in the subtitle (i.e., after a colon). For example, “Referral to the NHS Diabetes Prevention Programme and conversion from non-diabetic hyperglycemia to type-2 Diabetes Mellitus in England: a matched cohort analysis”

***Title has now been changed to “Referral to the NHS Diabetes Prevention Programme and conversion from non-diabetic hyperglycaemia to type-2 Diabetes Mellitus in England: a matched cohort analysis”***

3)      The data availability statement needs revision. Given the use of CPRD data please consider the following statement or similar: ‘Requests for access to data from the study should be addressed to [cprdenquiries@mhra.gov.uk](mailto:cprdenquiries@mhra.gov.uk). All proposals requesting data access will require approval from CPRD before data release.’
***Done.***

4)      Abstract:
a)      Please combine the Methods and Findings into one section “Methods and Findings”
b)      Please include the important dependent variables that are adjusted for in the analyses.-

***The dependant variables have been added (Page2,line 18-21)***
c)      Please ensure that all numbers presented in the abstract are present and identical to numbers presented in the main manuscript text.
d)      Please quantify the main results (please present both 95% CIs and p values).

***Amended***
e)      In the last sentence of the Abstract Methods and Findings section, please describe the main limitation(s) of the study's methodology.

***Sentence now added “As it is an observational study, a limitation would be the ability to making any causal inference” (Page 2, line 30-31)***

5)      Please delete the “Research in Context” section

***Amended***

6)      At this stage, we ask that you include a short, non-technical Author Summary of your research to make findings accessible to a wide audience that includes both scientists and non-scientists. The Author Summary should immediately follow the Abstract in your revised manuscript. This text is subject to editorial change and should be distinct from the scientific abstract. Please see our author guidelines for more information: <https://journals.plos.org/plosmedicine/s/revising-your-manuscript#loc-author-summary->

***This has now been added. (Page 3)***

7)      Did your study have a prospective protocol or analysis plan? Please state this (either way) early in the Methods section.

***The Study had an analysis plan, which is now included in the text. (Page 5, line 22)***
a)      If a prospective analysis plan (from your funding proposal, IRB or other ethics committee submission, study protocol, or other planning document written before analyzing the data) was used in designing the study, please include the relevant prospectively written document with your revised manuscript as a Supporting Information file to be published alongside your study, and cite it in the Methods section. A legend for this file should be included at the end of your manuscript.

***Supplementary File added***

b)      If no such document exists, please make sure that the Methods section transparently describes when analyses were planned, and when/why any data-driven changes to analyses took place.
c)      In either case, changes in the analysis-- including those made in response to peer review comments-- should be identified as such in the Methods section of the paper, with rationale.

***Supplementary File added***

8)      For additional context, please describe how the NDPP works and how the referral system is structured.

***We have now added further details on how the NDPP works and how the referral system is structured. (Page 4, lines 18-28)***

9)      Your study is observational and therefore causality cannot be inferred. Please remove language that implies causality, such as “impact,” “effect” or “reducing the overall numbers.” Refer to associations instead.-

***Thank you, we have made changes to make that clearer, wherever possible. We also acknowledge this in the limitations section.***

10)     Given the limitations of the study design, in the Introduction, please remove descriptions such as “rigorous estimate of ….”
***Thanks, this has now been removed.***

11)     The terms gender and sex are not interchangeable (as discussed in <http://www.who.int/gender/whatisgender/en/> ); please use the appropriate term.

***We have now used the term “sex” throughout the manuscript.***

12)     Please ensure that the study is reported according to the STROBE and include the completed STROBE checklist as Supporting Information. Please add the following statement, or similar, to the Methods: "This study is reported as per the Strengthening the Reporting of Observational Studies in Epidemiology (STROBE) guideline (S1 Checklist)."

***This is now included (Page 5, lines18-19)***

a)      When completing the checklist, please use section and paragraph numbers, rather than page numbers.-

***Checklist completed***

13)     Did you adjust for ethnicity and deprivation status based on location or patients’ addresses?-

***No adjustment for ethnicity was carried out, deprivation status was based on location***

14)     Please move “Ethical approval” to the Methods section-

***“Ethical Approval” has now been moved (Page 8, lines 21-30)***

15)     Results:
a)In the results, please ensure p<0.0001 is revised to p<0.001-

***Thanks, this has now been changed***
b)Please quantify the main results with both 95% CIs and p values

***Thanks, this has now been added***

16)     Please define all abbreviations in Tables and Figures e.g., NDH, BMI, SD, T2DM

***Abbreviations has been now added to the tables and figures***

17)     Please define S(t) on the y-axis of Figure 2-

***This is the survival function, is the probability that the time of conversion to T2DM is later than the specified time t. This has now be added as a footnote to the respective graph.***

18)     For the survival curve in Figure 2, please show the numbers at risk at regular intervals below the x axis.-

***We feel this may make the graph to busy so instead we added a footnote where we present these numbers at 12, 24, 36 and 48 months.***

19)     In Figure 2, please show the axis beginning at zero. If this is not possible, please show a break in the axis.-

***Thank you, we did not previously to better demonstrate differences in the trends. The Y axis now is 0 to 1.***

20)     Please remove subheadings in the Discussion section, and present and organize the Discussion as follows: a short, clear summary of the article's findings; what the study adds to existing research and where and why the results may differ from previous research; strengths and limitations of the study; implications and next steps for research, clinical practice, and/or public policy; one-paragraph conclusion.-

***Thank you for the suggestion, we have now structured the discussion as per above suggestion and implications and next step also added.***

21)     Please remove the ‘Funding,” “Data sharing,” and “Completing interests” statements from the end of the main text. In the event of publication, this information will be published as metadata based on your responses to the submission form.

***This has now been removed***
22)     References:

a)      Please select the PLOS Medicine reference style in your citation manager. In-text reference call outs should be presented as follows noting the absence of spaces within the square brackets, e.g., "... services [1,2]."
b)      References should have six names before et al.
c)      Please ensure that journal name abbreviations consistently match those found in the National Center for Biotechnology Information (NCBI) databases. <https://journals.plos.org/plosmedicine/s/submission-guidelines#loc-references>.
 ***Reference format has been amended.***

Comments from the reviewers:

Reviewer #1:
**Minor comments**
1) In the Introduction section, the last statement states that the study assesses the impact of DPP in reducing the conversion of NDH to diabetes (incidence)-diabetes should be specified as type 2 diabetes (T2DM).

***Thank you, this has now been amended. (Page 4-line 38)***

2) In the Introduction section, it would be interesting to provide information on the number of people referred to the NDPP, how many complete the program in general. Furthermore, in the discussion, it states that the NDPP is effective in reducing weight and HbA1c, can the authors include the magnitude of the effects? What is the drop out rate?

***Thank you. This has now been added (Page 4,line 28-30) (Page 10-line 6-8)***

***The dropout rate varies at different stages, this is shown in Figure 1 by Valabhji et al in their publication on “Early Outcome from the English National Health Services Diabetes Prevention Programme”, which we cite in our work.***

3) In the Methods section, the cohort defined NDH using Read code for Glucose Tolerance Test abnormal-wouldn't this include people with T2DM? Was this a typo?

***No, this is the case. For NDH people the glucose tolerance test would be abnormal, and when selecting samples anyone who had a T2DM read code before or on the date of NDH diagnosis would be dropped from the sample. Thus, it is a two-stage process, that ensures the sample will contain only those with a NDH diagnosis and not T2DM diagnosis.***

4) In Figure 2, please define the abbreviation "stpm2"

***Apologies for this omission. Stpm2 is a user-written STATA comment to fit flexible parametric survival models that allows for reporting of average survival curves, rather than curves for the average person. This was already stated in the statistical analysis section, but we have also added it to the Figure 2 footnote as well.***

Major comments
1) The results showed that individuals treated with metformin had a higher risk of developing T2DM. Is there concern of misclassification whereby individuals with metformin prescriptions likely have a T2DM diagnosis? Should individuals with prescriptions for anti-diabetic agents be excluded from entering the cohort to avoid including those with T2DM? Similarly, did the study investigators considered individuals treated with weight loss agents such as GLP-1RA?

***There is evidence that metformin is also used as a drug to prevent or delay the onset of T2DM in those who are at high risk of diabetes.* *(Metformin for Prediabetes. JAMA. 2017;317(11):1171. doi:10.1001/jama.2016.17844). In the context of our research question, metformin is a measured confounder, and controlling for it is essential. Excluding cases prescribed metformin, i.e. those at the highest risk of developing T2DM, is problematic, since we will be excluding the target population of the intervention we aim to evaluate. We did not consider treatment with weight loss agents, since numerous over the counter options are available, thus we decided we could not reliably measure through prescriptions.***

2) In relation to comment #1, should diagnosis of T2DM be also defined as prescription of an antidiabetic agent in addition to the use of Read codes?

***In previous work we have found that T2DM (diabetes in general) is very well recorded in primary care, due to the Quality and Outcomes Framework (QOF) through which management of diabetes (both types) is financially incentivised and thus very well recorded. In the past there was not a single case in hundreds of thousands of diabetes patients who were treated for diabetes without a relevant diagnosis.***

3) I am concerned that the comparison group consisted of individuals from a non referring practice. Is the care provided by these centers different compared to the care provided by centers that refer patients to the NDPP?

***It should not be, again management of T2DM takes place under the QOF umbrella, so the processes are the same. Naturally, there may be variation in quality of care, but that is irrespective of participation to the intervention (i.e. practices were not self-selected to participate, rather they were rolled in as whole regions). Thus the reviewer has a point, but it should be random error at the practice level, which we try to adjust through the multi-level analyses. In addition, we do conduct sensitivity analyses with within-practice matching to overcome this, but that introduces a different type of bias at the patient level, perhaps, as explained in the paper. (Appendix-Figure 2, Table 4 & 5).***

4) Socioeconomic status (SES) information was not included in the complete case analysis which is a limitation since it was not available for individuals registered in non-English practices (which many were non referring centers). SES is a significant confounder that was not accounted for (i.e. people with lower SES likely have a higher risk of developing T2DM and may be less likely to participate in the NDPP).

***This is a valid concern. As previously mentioned, this would have been a major issue if practices were self-selected, which is not the case. Thus, the characteristics across referring and non-referring practices are similar, region aside. Nevertheless, we did conduct sensitivity analyses that included both practice and patient deprivation, with multiple imputation. Results were comparable and these are reported in, the appendix. (Table 4)***

5) Given that people who participate in the NDPP may have healthier behaviors compared to people who do not participate, can the analyses adjust for proxies of healthy behavior such as physician visits/year, or vaccination uptake?

***Again, this is a valid concern and we are limited by the information on healthy behaviours included in the primary care records. The reviewer mentions two potential proxies, which are interesting. Physician visits are potentially problematic since they may indicate poor health, rather than proactivity in seeking help (although they would probably indicate the latter in some cases). Vaccination uptake, we imagine the reviewer means in the context of COVID-19, is not useful, since our study period ended before vaccines were available. Any other type of intervention in this population will not be relevant to all patients, and thus could not be used as a proxy. However, this is the main driver of our choice of main analysis. The between practice matching aims to address exactly that limitation, to reduce bias introduced by differences in motivation and behaviours, in referred and non-referred patients.***

Reviewer #2: Thanks for the opportunity to review your manuscript. My role is as a statistical reviewer, so my review focuses on the study design, data, and analysis presented in the manuscript. I have put general comments first, and followed these with queries relevant to a specific section of the manuscript (with a page/paragraph reference).

The study used an interesting matching approach - using the staggered nature of the program introduction, they matched (stratified by available follow-up time) practices that begin referrals at a particular time-point to a practice that hadn’t begun referrals (based on what I think is number of patient identified with NDH), and then within this practice-matched pool, exactly matched patients from one practice to the other (based on age (3 year distance), sex, and date of diagnosis (1 year window). This has the strength of making the unexposed patients contemporary   with the exposed (those referred to the DPP) – this is overall a good approach to matching and I’m impressed they were able to do the exact matching (this never seems to work for me). The resulting selected cohort appears to be matched pretty well for most variables. A parametric survival model with shared frailty (practice) is used for the main analysis – I had a few queries about this but on the whole is a good
approach given that it sounds like the provision of referrals sounds like it was made available at a practice level. The results are presented in both relative (HR) and absolute terms which is helpful.

Typically the ‘case/control’ terminology is used to describe outcomes (T2DM incidence/no incidence), rather than exposures (exposed/unexposed, referred to NDH/not referred to NDH). I would consider updating the terminology throughout the manuscript, using referred to NDH/not referred is probably the clearest way to convey the information.

***We thank the reviewer for his/her overall positive verdict. Thank you, that is a valid point and we have made changes throughout in the terminology to make the paper easier to read.***

P5, Paragraph 2. Are there differences in the regions covered by the two databases? Are the two databases generalizable to the population served by NHS?

***CPRD GOLD collects data from the four countries in the UK, with around 30% of the data contributed by practices in England. CPRD AURUM is mostly practices in England with practices in Northern Ireland contributing data from 2019. Previous studies have shown that both databases are representative of the English population in terms of deprivation as well as age and gender, and fairly representative in terms of geography. Relevant papers:***

[***https://academic.oup.com/ije/article/44/3/827/632531***](https://academic.oup.com/ije/article/44/3/827/632531)

[***https://academic.oup.com/ije/article/48/6/1740/5374844***](https://academic.oup.com/ije/article/48/6/1740/5374844)

[***https://bmjopen.bmj.com/content/8/2/e020738***](https://bmjopen.bmj.com/content/8/2/e020738)

P5, Paragraph 5. When I went to look at clinicalcodes.org, it was mainly content about steroid use in baseball. I'd consider dropping the 'www' so that people don't go directly to that (incorrect) URL, and include the .man.ac.uk URL in the reference list.
***Thank you. It is changed now. (Page 5, line 3). The address was registered to a person rather than the UoM and we could not continue renewing, thus it was picked up by spammers.***

P6, Paragraph 1. For the study period where matching was used, what was the distribution of referrals like? I was interested in what proportion of the total sites was non-referring, how many made 2 or more referrals, and how many made 1-19.

***Out of the 2207 practices 849 had 0 referrals and 500 had referrals in the range of 1-19 and 858 had over 20 referrals***

I was unclear what the variable 'NDH registers' meant, is this the number of patients identified as having NDH?
For the propensity score matching of sites, was there evidence of common-support?

***Yes, NDH-registers are the number of patients who were identified as having NDH. We have made this clearer at first mention of the term (Page6, line 22-23). There was some evidence of overlapping PS in the two groups, but it was not ideal due to the nature of 1 to 1 matching – i.e. all practices are matched to nearest neighbour without replacement (see image below). Although not ideal we still think this is the best approach, complemented by numerous sensitivity analyses, by removing referring practices that have little in common with non-referring practices. We also performed the 1 to N matching sensitivity which is largely in agreement with our main analyses. There we set a very low caliper to ensure close matching. No analysis was perfect, each of the analyses we conducted came with different limitations, but our wide range of different approaches arrives at similar results.***

P6, Paragraph 2. Were the covariates included in the form seen in appendix table 2/3?
The missing-as-indicator approach relies on the data being 'missing-completely-at-random'. In particular, for unbiased estimates, that the presence of a missing value is not correlated with any other of the covariates. In the sensitivity analyses, a version of the analysis with multiple imputation is used and these general shrink the main estimate back to a null (HR=1) effect. This suggests that the missingness can't be considered MCAR. What was the rationale for the missing-as-indicator approach to be used for the main analysis given that the MI analysis is robust to data that is at least missing-at-random?

***This is another good point. We a-priori decided on the complete case approach because we would have to impute very large percentages of the data, especially for HbA1c and patient-level deprivation (completely missing for non-English practices). We do see similar patterns with the multiple imputation approaches, although as the reviewer rightly says, in some case that is NS or even very close to null.***

P7, Paragraph 2. Was the flexible parametric survival model used in both the main analysis and for the analysis for average survival curves within groups? Where it was used, what was the form of the model and how was it selected (e.g. number of knots)?

***We used a parametric survival model for the main analysis (streg with a Weibull survival distribution in Stata), which was in agreement with a standard Cox regression – but much faster allowing us to run numerous models and bootstrap the error. Stpm2 was the flexible parametric survival model, but that was only used to obtain the graph, as previously mentioned. The main reason we did not use this command in the analysis is that it could not model random effects for practices, which we established was quite important. The full code for stpm2 was:***

***bootstrap, reps(1000) cluster(mcluster): stpm2 case gender _Iagegrp_2 _Iagegrp_3 _Iagegrp_4 _Iagegrp_5 _Iagegrp_6 _Iagegrp_7 _Icholcat1_1 _Icholcat1_2 _Icholcat1_3 _Icholcat1_4 _Icholcat1_9 _Ismoking_2 _Ismoking_3 _Ismoking_9 _IBMI_cat_1 _IBMI_cat_3 _IBMI_cat_4 _IBMI_cat_9 _ICCI_score_1 _ICCI_score_2 _ICCI_score_3 Dep Met _ISystolic__2 _ISystolic__3 _ISystolic__4 _ISystolic__9 _IDiastolic_2 _IDiastolic_3 NDH_Ref, scale(hazard) df(4) eform***

***In short, we bootstrapped in clusters (to ensure case and the respective control practice(s) was (were) selected as a unit), and opted for four degrees of freedom as suggested by the command (one to four is suggested by the command): “Option df() specifies the degrees of freedom for the restricted cubic spline function used for the baseline function and it must be between 1 and 10, but usually a value between 1 and 4 is sufficient, with 3 being the default”. We considered all this to be unnecessary information for obtaining a figure, in an already very complex paper methodologically.***

What was the rationale for using the bootstrapping? Was it a purely cluster-based bootstrap (i.e. each site was selected in each bootstrap sample with all patients), or was it a two-stage bootstrap (bootstrapping both site and patients within each site).

***The rationale for bootstrapping was to obtain more robust standard errors. It was purely a cluster-based bootstrap, as shown in the previous comment.***

P7, Paragraph 3.
How was the number of imputations for the MI sensitivity analysis decided?

***Decisions on this are almost always arbitrary and driven by computational demands. 10 is considered adequate and it’s a nice round number! Apologies for not being too scientific on this.***

It would also be nice for detail about the rationale for each of these sensitivity analyses (just a few sentences) in the appendix) as there is detail for some of these sensitivity analyses but not others.

***We have explained the sensitivity analyses in the main paper, and there is no rationale regarding the sensitivities in the appendix, as far as we can see (there was a mention of sensitivity, but that did not relate to what we described as sensitivity analyses and the wording has now changed to avoid confusion). We feel the description in the main paper is adequate. There is no particular rationale in most cases, besides the obvious, e.g. 1 to N rather than 1 to 1 practice matching to include more practices but potentially include practices that are too different to be matched.***

P9, Paragraph 6. The limitation of making a causal inference from observational data should be directly acknowledged here.

***Thank you, we now included there as well (Page 10, line 2)***

Appendix Fig 1. This is a very clear figure to follow - is it possible to have a similar (separate) figure for the exclusion of sites?

***Thank you for your kind words. This is a very good suggestion and we have now included it for the 1 to 1 and the 1 to N practice matching. (Figure 2 & 3 in the Appendix)***

Appendix Table 2/3. Is it possible to get a column with standardised difference for each variable? Most of these look like a good match but it's hard to get a sense for variables with a high SD like follow-up from NDH diagnosis in Table 2.

***Thank you for the suggestion. We are not sure what the reviewer means exactly. Is it standardised mean difference? We have never seen SMD used in this context (mainly in meta-analysis to pool measures in different scales) and we are not sure what it would add and how it would be interpreted. Considering the current complexity of the table, we would prefer to leave it as it is, respectfully.***

Reviewer #3: General Comment:  This is a potentially important paper because, despite the hope and potential for wide-scale diabetes prevention programmes though behavioural support, the effectiveness of such approaches implemented in the real world have never been established.  This paper is generally well written and the analytic design seems appropriate.  But the paper falls far short in providing definitive conclusions due to lack of clarity and comprehensiveness in the analysis.  My specific comments/suggestions are the following:
1.      Study denominator selection:  The abstract refers to "complete case analysis" and then we never hear of that again (unless I missed it) . Exactly what defined a complete case, why was this approach chosen, and what approaches were taken to understand the characteristics of the non-complete cases, and most importantly, what are the implications (on quantitative estimates) of leaving them out? These are issues that should be included in the methods, results, and particularly discussion section, where it is lacking. (At present the discussion spends a lot of time talking about nuances of the matching decisions, which is comparatively trivial.)

***Thank you for highlighting this. In the “Covariates” section we say:***

***“Where levels of missingness were considered to be high for a biological parameter (above 50%), it was not included in the main analyses (HbA1c). Socio-economic information was not included in the complete case analysis due it not being available for patients registered in non-English practices. In the multiple imputation analyses, all covariates were included irrespective of levels of missingness. More information on extraction of the covariates relative to the index date and categorisation, where relevant, is provided in Appendix”. In the same section we describe all the available covariates. Thus all these were included in the model, except for the two socio-economic level covariates (patient and practice level) and HbA1c. We feel this is clear enough and perhaps the reviewer missed it.***

***Regarding the implication of leaving out these covariates, that is a very good question that is very difficult to answer. We have tried to answer that question through some of the sensitivity analyses, e.g. multiple imputation. There is broad agreement in the findings, but that is why the sensitivity analyses, including different matching approaches, are important and we are trying to discuss their strengths and limitations. We do discuss this as limitation and also explain that one of the sensitivity analyses addresses that to some extent: “Another possible limitation is not being able to include HBA1C in our model due to high levels of data missingness – although we included it in the multiple imputation analyses”.***

2.      Effects of differential outcome measurement:  There is no reporting of the potential for differential measurement that could affect detection biases.  As this study is based on passive rather than active determination of incidence (ie., letting it happen and be reported through primary care, rather than standard measurements), there should be some assessment of whether intervention participants received more HbA1c measurements than non-participants. Table 1 actually suggests the opposite of that is the case, but that is % with values at all.  Would also be helpful to see whether there is variable numbers of HbA1cs.  This directly affects outcome ascertainment.

***Thank you for raising this issue. This is a valid point, and this study is dependent on the quality of recording in primary care and current processes. So, although there are guidelines for relatively frequent assessment of NDH patients, to examine if they have progressed to T2DM, this is dependent on the patient agreeing to the assessment. The potential bias the reviewer implies, is that referred cases are more proactive in managing their health care needs, and thus they may be more frequently visiting primary care, and thus be diagnosed with T2DM earlier. That is not an implausible risk, but one which would reduce the observable “effect” of the intervention. Thus our effectiveness estimate would be conservative. Table one lists the follow up of patients within the database until exit (of the general practice of the individual) or until conversion to T2DM and does not report on numbers of visits to general practice for each. We had explored variation in interactions with primary care in the two groups but did not report it previously (because the paper was busy enough and the difficulty in reporting a meaningful measure of interactions with primary when follow up varies between groups – and problematic to include in a time-to-event analysis). These were similar in the two groups, considering the somewhat longer follow up for referrals (see table 1 and the figure below). Nevertheless, we discuss this as a limitation in the relevant section:***

***“Finally, referred and non-referred people may have different health seeking behaviours, with referred people diagnosed with T2DM earlier due to higher levels of interaction with primary care. In that case we would be underestimating the impact of the intervention.”***

***Going back to HbA1c, there is some variability but the test information is missing for the vast majority of the cohort, thus it is not information that can be accounted for in the analyses in a meaningful way.***

**Figure 2: Mean of total interactions with primary care in each QOF year**

3.      Putting this paper in context.  This discussion, and to a lesser extent the introduction as well, does a poor job putting this paper in context of the other literature, which is enormous in other domains (RCTs; community translation trials, CE modelling, etc.).  Neglecting more comment on this ends up underselling the

***Thank you for this. This is a pretty generic comment that we are not sure how to deal with. The paper is around 4000 words with 30 references (many of which are RCTs of interventions or systematic reviews). It was not our aim to provide a systematic review of evidence on NDH intervention (one already exists which we cite), but to evaluate the NHS DPP.***

4.      Introduction section:  The first 2 paragraph are a bit clunkily written.  It goes from describing the problem, then to the evidence for prevention, then about what NDH is, then back to evidence for prevention. At the same time, the introduction does not provide the overall evidence context very well.  There is far more that precedes this paper and makes it important than the handful of RCTs that are referenced.  Suggest better focus and a re-write of the introduction.

***Pleas see our previous response. Our introduction describes the problem, then summarises evidence for prevention, then describes what NDH is and its uptake – then we explain what we plan to do in this paper. We do not go back to evidence for prevention.***

5.      NDH Case selection:  Page 5, 3rd paragraph:  Says this group explored how well NDH codes identify NDH but then stops there and doesn't tell us what they found, and if they are any good.  In most systems, read codes or ICD coding is horribly non-sensitive and probably biased towards selecting high risk participants.  Did that happen here?

***This is a valid point and we have rephrased for clarity. The definition of NDH has changed over time, and so has prevalence. We cannot really evaluate how many NDH cases are not recorded in the EHRs we use, so there is always a risk that this is not the full population. In the context of whether the intervention works, however, this is a minor issue, unless we are contained to a very special subpopulation, which is not the case (as evidenced by the prevalence we observe and the conversion rates – which are in agreement with the literature, hence we are not seeing the high-risk cases alone).***

6.      Matching process and selection:  The matching approach seems solid, and in general, doing this at practice rather than individual level seems a strength; however, wo questions come up:
    a.  Was measurement frequency (ie., A1c) considered as a variable in the matching process to limit the potential selection or detection bias?

***Thank you for your positive assessment. With observational studies there are numerous limitations, so we tried different designs with different strengths and limitations, from which we broadly received the same answer (albeit of varying levels of effectiveness). Unfortunately, HbA1c was missing for most referred and non-referred patients, hence we could only use it in the multiple imputation analysis. For that reason, we did not include it in the patient matching – and we would have, this is a very good suggestion. This is related to a previous point you raised, we have now added as a limitation.***

    b.  Does the use of practice based matching mean that if a given practice is more involved in NDPP referral, that they are also differentially involved in detection?

***This is a reasonable concern and an issue we tried to address in the between-practice matching by excluding practices with low referral numbers (1 to 20). The intervention hadn’t been rolled out in the non-referring practices (yet; or at all for those not in England). There will be practice variability that we cannot fully capture and that is why we also report on the (much easier to conduct) within-practice matching approach. Although that approach does not suffer from practice variability concerns, the major risk of bias there is the health seeking behaviour variability within the practice with motivated people opting to be referred. Overall, the broad agreement across all these approaches gives us confidence.***

7.      Analytic approach: following form comment 1 above, how is complete case analysis defined here, who exactly is included and excluded. All of this deserves good description in methods, showing implications of this approach in the results, and discussing it in the discussion.

***This relates to the first point raised. The inclusion and exclusion criteria are described in figures 2-4 in the appendix Regarding the implications of not including the three covariates previously mentioned (See response in point 1), we can only evaluate through the multiple imputation sensitivity analyses (and that was the whole point of them).***

8.      Referral definitions:  Page 5, paragraph 4 describes all the different referral codes and participation levels apparently.  Given that this is the crucial exposure, it would be nice to see what these codes represent, how they are defined, and how many.  It appears that ALL of these groups considered as "cases"?   Please clarify.

***Most of the information is already there and perhaps it was missed:***

***“Participants were considered as referred to the programme if they were associated with one of the following codes: 679m000/EMISNQDI236-NHS DPP not completed, 679m100 NHS DPP completed,679m200 NHS DPP started,679m400/ EMISNQRE591 Referral to NHS DPP, EMISNQAT50-NHS DPP attended, EMISNQDI236-NHS DPP not attended).”***

***How many is not really central to our research question and did not feed into any analytical decisions (besides dissuading us from analysing refusals, since so few were recorded). However, we do report on referral declined in Table 1 in the appendix and we report relevant information in the table below:***

|  | **NDH** | **Referred** | **Attended** | **Completed** | **Referred Declined** |
| --- | --- | --- | --- | --- | --- |
| **01/04/2016** | 354713 | 9930 | 1456 | 229 | 2736 |
| **01/04/2017** | 429765 | 43022 | 8257 | 1045 | 16187 |
| **01/04/2018** | 529361 | 93055 | 19275 | 2926 | 39545 |
| **01/04/2019** | 618967 | 143388 | 36812 | 6089 | 67117 |
| **01/04/2020** | 619536 | 150461 | 37876 | 6552 | 69847 |

9.  Additional minor point; calling these "cases" is awkward in a prospective trial emulation because they are not really cases of disease, they're actually non-cases and participants, some of whom become cases;  I would encourage different terminology).

***We accept this criticism, we now discuss the two groups as “referred” and “not referred”.***

10.     Description of findings.  Several analyses would improve our ability to interpret the findings of this study, including:
   a.   Presentation of numbers by each level of referral group (completed, vs not, etc.).

***Very good point and what we were planning to do originally, but the data simply is not there for completion. We have expanded the limitations section to explain why we could not deliver this.***

   b.   A table comparing characteristics of the non-NDH, NDH, referred without NDH, referred and completed, attended, etc.  ie., who are these people?

***This is of interest perhaps but not central to our research question. Our main analyses excluded cases without an NDH diagnosis, since the intervention was only available for people with NDH. However, we conducted a sensitivity analyses, included these “non-NDH” interventions and the results were almost identical. As previously mentioned we do not have reliable information on attendance and completion, so we cannot report such a table. But we have now added a table below to summarise key characteristics of NDH and “non-NDH” referrals (referred and non-referred NDH comparisons are already presented).***

|  | **All** | **referred** | **Non-NDH Referrals** |
| --- | --- | --- | --- |
| **All** | 41,056 | 30,508 | 10,548 |
| **Males** | 19826.0(48.3) | 14756.0(48.4) | 5070.0(48.1) |
| **Females** | 21230.0(51.7) | 15752.0(51.6) | 5478.0(51.9) |
| **Age (years)NDH diagnosis date** | 62.0±12.0 | 61.9±11.9 | 62.3±12.3 |
|  |  |  |  |
| **Age group (Years) NDH diagnosis date** |  |  |  |
| **18-34** | 477.0(1.2) | 338.0(1.1) | 139.0(1.3) |
| **35-44** | 2766.0(6.7) | 2064.0(6.8) | 702.0(6.7) |
| **45-54** | 8067.0(19.7) | 6024.0(19.8) | 2043.0(19.4) |
| **55-64** | 11727.0(28.6) | 8809.0(28.9) | 2918.0(27.7) |
| **65-74** | 11481.0(28.0) | 8576.0(28.1) | 2905.0(27.5) |
| **75-84** | 5681.0(13.8) | 4088.0(13.4) | 1593.0(15.1) |
| **>=85** | 857.0(2.1) | 609.0(2.0) | 248.0(2.4) |
| **BMI (kg/m2)** | 31.0±6.5 | 31.2±6.6 | 30.4(6.2) |
| **BMI Categories** |  |  |  |
| **<18.5** | 204.0(0.50) | 156.0(0.51) | 48.0(0.46) |
| **18.5-25** | 3877.0(9.4) | 2682.0(8.8) | 1195.0(11.3) |
| **25-30** | 8697.0(21.2) | 6134.0(20.1) | 2563.0(24.3) |
| **>=30** | 13301.0(32.4) | 10004.0(32.8) | 3297.0(31.3) |
| **Missing** | 14977.0(36.5) | 11532.0(37.8) | 3445.0(32.7) |
| **Charlson comorbidity score** |  |  |  |
| **None** | 23307.0(56.8) | 17146.0(56.2) | 6161.0(58.4) |
| **1 to 2** | 10914.0(26.6) | 8580.0(28.1) | 2334.0(22.1) |
| **3 to 4** | 3892.0(9.5) | 2831.0(9.3) | 1061.0(10.1) |
| **>4** | 2943.0(7.2) | 1951.0(6.4) | 992.0(9.4) |
| **Cholesterol (%)** |  |  |  |
| **<3** | 687.0(1.7) | 501.0(1.6) | 186.0(1.8) |
| **3 to 4** | 5525.0(13.5) | 4116.0(13.5) | 1409.0(13.4) |
| **4 to 5** | 10053.0(24.5) | 7155.0(23.5) | 2898.0(27.5) |
| **5 to 6** | 8601.0(21.0) | 6067.0(19.9) | 2534.0(24.0) |
| **>=6** | 5068.0(12.3) | 3570.0(11.7) | 1498.0(14.2) |
| **Missing** | 11122.0(27.1) | 9099.0(29.8) | 2023.0(19.2) |
| **Depression** | 10535.0(25.7) | 8303.0(27.2) | 2232.0(21.2) |
| **Smoking Status** |  |  |  |
| **Current Smoker** | 13375.0(32.6) | 10043.0(32.9) | 3332.0(31.6) |
| **Ex-smoker** | 11116.0(27.1) | 7722.0(25.3) | 3394.0(32.2) |
| **Never smoker** | 15996.0(39.0) | 12376.0(40.6) | 3620.0(34.3) |
| **Missing** | 569.0(1.4) | 367.0(1.2) | 202.0(1.9) |
| **Type 2 Diabetes** | 2438.0(5.9) | 2002.0(6.6) | 436.0(4.1) |
| **Metformin** | 1264.0(3.1) | 983.0(3.2) | 281.0(2.7) |
| **Systolic Blood Pressure(mm Hg)** | 135.3±14.3 | 135.4±14.2 | 134.9±14.5 |
| **Diastolic Blood Pressure (mm Hg)** | 79.4±9.0 | 79.3±8.9 | 79.6±9.3 |
| **HBA1C (mmol/mol)** | 43.4±4.0 | 43.5±4.1 | 43.2±2.9 |
| **% with HBA1C values** | 13687.0(33.3) | 11681.0(38.3) | 2006.0(19.0) |

   c.   Presentation of incidence rates of conversion (events per 1000 per year) for appropriate external comparison.

***We do not analyse per year, this is not a typical cohort study on which to report incidence rates of conversion (we presented these in our previous paper: Ravindrarajah R, Reeves D, Howarth E, Meacock R, Soiland-Reyes C, Cotterill S, et al. Epidemiology and determinants of non-diabetic hyperglycaemia and its conversion to type 2 diabetes mellitus, 2000–2015: cohort population study using UK electronic health records. BMJ open. 2020;10(9):e040201. doi: 10.1136/bmjopen-2020-040201). We quantify conversion risk in the two groups in the results section and we also present Figure 2. From these events per 1000 cases can easily be obtained.***

***“The rate of conversion to T2DM was lower in cases compared to controls with a HR of 0.80 (95% CI: 0.73, 0.87) (Table 2). Non conversion (‘survival’) at 24 months since referral was estimated at 89.9% (95% CI: 89.5% to 90.4%) for controls and 91.8% (95% CI: 91.2% to 92.3%) for cases. At 36 months it was 84.6% for controls (95% CI: 83.9% to 85.4%) and 87.3% for cases (95% CI: 86.5% to 88.2), respectively. The difference in conversion rates at 36 months was -2.7% (95% CI: -3.7% to -1.7%). Average survival plots across the two groups are presented in Figure 2”***

   d.   Showing comparison of rates across these groups, eligible vs referred vs attended, etc..

***Please see response to point 10b***

   e.   For the subset with serial HbA1c measurements, show those trends comparing participants and matched controls.

***This point has been discussed before. This information is largely missing and very few individuals have numerous measurements, to be analysed separately.***

11.     Minor point - the association of obesity with conversion and diabetes risk is usually curvilinear.  Why not show the risk ration with class II and class III obesity, given that there is likely ample sample size.

***That is why we analyse as a categorical variable, and observe a curvilinear relationship. The cut-off points could have been different but this is not really central to our research question.***

12.     Discussion section:  This section really falls short in doing 3 things:  1) Explaining the context of this paper in the broader literature, so that the reader knows how potentially important this study is;  2)  Addressing the potential biases that could be at play in a quasi-experiment like this, and more importantly, explaining what the most likely implications are on findings;  3) Discussing what this study really means for practice and policy.
***It should perhaps be noted that other reviewers felt that it was well written. The reviewer argues the discussion fails on practically everything a discussion section should be doing. Rather than completely rewrite/rephrase to include the same information, considering the size of the paper (4000 words), we answer each point of critique below.***

***The context of the paper in the broader literature: This is primarily addressed in the introduction, but in the discussion dedicate 450 words to discussing in the context of the literature (From “Our findings suggest…” to “…characteristics of the people who attended the programmes”)***

***Addressing the potential biases: We cannot address the potential biases in such an observational study – not all of them with the same design at least. That is why we report on a numerous different sensitivity analyses. Some of the limitations of the study cannot be overcome completely, unfortunately. We have been working on this project for well over three years, but, sadly, we are not magicians. The strengths and limitations section where we iterate issues we previously described in the methods section is 508 words in length.***

***Implications of findings and what it means for practice and policy: This is an evaluation of a real country-wide intervention, thus the implications of our findings are somewhat obvious: it works and please continue. This is the simplest part of the paper, arguably and we conclude the paper by saying:***

***“The NDPP appears to be an effective intervention, at least in the short to medium term. Individuals who were referred to the NHS DPP by primary care physicians were less likely to develop T2DM compared to those who received usual care Although we observed lower levels of associations compared to what has been observed in randomised clinical trials, we examined the impact of referral, rather than attendance or completion of the intervention. It will be important to explore whether the association of the programme varies across population subgroups, as well as the long-term effects of the programme. Finally, further work is needed to examine the long-term effects of the programme, and to examine if the programme delays or prevents T2DM”.***

***Anything else when it comes to practice and policy, e.g. cost-effectiveness, is beyond the scope of this paper and will be presented in separate work.***

Reviewer #4: Very important study on the effectiveness of referral to the programme at reducing conversion of NDH to T2DM. I dont have sufficient expertise to comment on the methodology, but the overall rationale and discussion of results were well-elaborated.
***Thank you for your positive assessment.***
