# Supplementary material for: Referral to the NHS Diabetes Prevention Programme and conversion from nondiabetic hyperglycaemia to type 2 diabetes mellitus in England: A matched cohort analysis
Source: PLoS Med. 2023 Feb 27;20(2):e1004177. doi: 10.1371/journal.pmed.1004177 (PMC9970065; doi:10.1371/journal.pmed.1004177)
Supplement: Attachment — Submitted filename: Response 2.docx [file pmed.1004177.s006.docx]

*I have reviewed the reviewer 3's comments and reply from the authors. However, I don't think the authors answered the initial questions raised by the Reviewer 3 satisfactorily, especially the selection bias due to coding and the participants' willingness to receive measurement, etc. This will impact the interpretation of the outcome of this study. Although the topic of this study is interesting and important, there do exist some limitations in the methodological part, and the authors didn't solve these problems in their 2nd round revision.
The authors should improve the display of the whole manuscript to make it more reader-friendly.*

**Response:** Thank you for your comments. The review is very brief and rather unclear, but we have done our best to try to address. The original third reviewer had commented on NDH case selection and the measurement frequency of HbA1c, and we think these are the points the new reviewer highlights. In terms of whether our responses in general are satisfactory, it is not made clear by the new reviewer as to what we can do to improve them. The study, as any observational study, comes with limitations about which we have been very clear, and we have tried to overcome to some extent through numerous sensitivity analyses. Thus, we are not clear as to what the new reviewer thinks can be resolved, considering the data limitations. The last comment is also unclear, and we are not sure what makes the paper not reader-friendly, so we can correct – the methods, unfortunately, are necessarily complex to try to minimise confounding, so we can obtain a robust result. For clarity, here are some of the previous points raised by original reviewer #3 and our responses.

| *5. NDH Case selection:  Page 5, 3rd paragraph:  Says this group explored how well NDH codes identify NDH but then stops there and doesn't tell us what they found, and if they are any good.  In most systems, read codes or ICD coding is horribly non-sensitive and probably biased towards selecting high risk participants.  Did that happen here?* | This is a valid point and we have rephrased for clarity. The definition of NDH has changed over time, and so has prevalence. We cannot really evaluate how many NDH cases are not recorded in the EHRs we use, so there is always a risk that this is not the full population. In the context of whether the intervention works, however, this is a minor issue, unless we are contained to a very special subpopulation, which is not the case (as evidenced by the prevalence we observe and the conversion rates – which are in agreement with the literature, hence we are not seeing the high-risk cases alone). |
| --- | --- |
| *6. Matching process and selection:  The matching approach seems solid, and in general, doing this at practice rather than individual level seems a strength; however, wo questions come up:     a.  Was measurement frequency (ie., A1c) considered as a variable in the matching process to limit the potential selection or detection bias?* | Thank you for your positive assessment. With observational studies there are numerous limitations, so we tried different designs with different strengths and limitations, from which we broadly received the same answer (albeit of varying levels of effectiveness). Unfortunately, HbA1c was missing for most referred and non-referred patients, hence we could only use it in the multiple imputation analysis. For that reason, we did not include it in the patient matching – and we would have, this is a very good suggestion. This is related to a previous point you raised, we have now added as a limitation. |

Comments from the original reviewers:

***Reviewer #1:***

*I have reviewed the revised manuscript and the responses to my comments. The authors have addressed all my comments and I do not have any further concerns. Thank you for involving me as a reviewer.*

**Response:** Thank you

**Reviewer #2:**
*Thanks for the revised manuscript and answers to my queries. Overall most of my original queries have been resolved in this revision. Just a few minor points below.

Generally you don't always get a perfect match of propensity scores, so the use of calipers is not itself problematic. Just judging from the summary statistics, there was a reasonable degree of common support.*

**Response:** Thank you

*For appendix 2/3 the suggestion was for standardised difference (between exposure groups), there is a good description of this in Austin 2009 (*[*https://doi.org/10.1002%2Fsim.3697*](https://doi.org/10.1002%2Fsim.3697)*). This is fairly straightforward addition and while I agree that the table is already complex, this would probably simplify some of that complexity to highlight that is very limited differences after the matching process.*

**Response:** Apologies for misunderstanding your point previously. We appreciate the suggestion, but the table is already too busy, as the reviewer indicates, and we are not convinced by the need for the additional column. The main reason being that this is easily estimable from what we report, and we would rather include the full characteristics of the two groups, rather than the standardised difference.

For Figure 2 I would consider inverting the y-axis (so it's is cumulative incidence of conversion) and limiting the range as well to visualise the differences more easily. If possible, labelling each group directly on the figure is probably the best way to indicate which line is Normal or DPP.
**Response:** Thank you, but what we report is standard visualisation for survival analyses, and inverting the y-axis may lead to confusion. It is good statistical practice not to limit the range, to better evaluate the differences in the context of the whole population, so that was a conscious choice too (we do not want to exaggerate the differences in the two groups). The lines are too close to overlay labels, under our 0-100 y-axis approach, and we think the legend is perfectly adequate – there is no confusion about which line is which. However, we are happy to take any of these points on board at the production stage, if the editor and production team, prefer the reviewer’s suggestions.

It looks like the ClinicalCodes repository still has some issues with the domain - could you just include these in the appendix? They are an essential part of the reproducibility of the study.

**Response:** Apologies, this has now been corrected, the link is <https://clinicalcodes.rss.mhs.man.ac.uk/> but the codes have also been provided in an additional supplementary file.
