# Supplementary material for: Referral to the NHS Diabetes Prevention Programme and conversion from nondiabetic hyperglycaemia to type 2 diabetes mellitus in England: A matched cohort analysis
Source: PLoS Med. 2023 Feb 27;20(2):e1004177. doi: 10.1371/journal.pmed.1004177 (PMC9970065; doi:10.1371/journal.pmed.1004177)
Supplement: Attachment — Submitted filename: Response 3.docx [file pmed.1004177.s007.docx]

After consulting the academic editor, the editorial team are concerned that some of the original issues raised by Reviewer #3 (on the R1 version) remain unresolved and require resolution before we can consider publication:

Relating to Reviewer #3, Comment 2 (R1 Version): It is the academic editor's understanding that, in Table 1, the rates with HbA1c values were much lower in the "referred to NDPP" group than in the "not referred to NDPP" group. The academic editor notes that this is quite strange and is curious to understand why. As reviewer #3 and the academic editor note, this observation is the opposite of what one would expect, and led reviewer #3 to raise concerns regarding detection bias. Although the academic editor acknowledges that the authors provided some explanation in their response (R2 version, e.g., more proactive health management by referred cases leading to earlier T2DM diagnosis), they do not find this to be sufficiently convincing. If this was the case, those referred to NDPP should pay more attention to glucose monitoring. Please provide further comment on this point.

**Response**: We have revisited this and the main driver appears to be country-wide variation across England, Wales and Scotland. Cases are only present in England (the programme was implemented in England alone during the study period) while controls are drawn from England, Scotland and Wales, with a relatively large number from Scotland and Wales. In the England only controls, we see 6,619 HbA1c measurements over 28,479, or 23% of patients with an HbA1c measurement, which is much closer to the 19% seen in cases. Thus, in short, HBA1c recording was better in Scotland and Wales. Matching across countries was essential, as previously mentioned, due to the universal introduction of the programme in England, but we have sensitivity analyses that attempt to address that (focusing on England controls only). We have also added this in the discussion (limitations section) to clarify the issue for readers.

*“HbA1c completeness levels also varied between those referred to the programme and those who did not, with more missing data for those referred, which on the surface appears counter-intuitive, but that was primarily driven by variation in recording across countries, with better recording in Scotland and Wales.”*

Relating to Reviewer #3, Comments 3 and 4: The academic editor feels that author responses to these comments (R2 version) did not address the issues raised and suggests that the authors reorganize the introduction and discussion sections to ensure clarity and to provide important context to aid reader comprehension. Please address past research and explain the need for and potential importance of your study. Indicate whether your study is novel and how you determined that. Please present and organize the Discussion as follows: a short, clear summary of the article's findings; what the study adds to existing research and where and why the results may differ from previous research; strengths and limitations of the study; implications and next steps for research, clinical practice, and/or public policy; one-paragraph conclusion.

**Response**: We have now revised the introduction and discussion to address this.

Please update the line numbering to be continuous throughout the manuscript (rather than restarting on each new page).

**Response**: Done.

Your study is observational and therefore causality cannot be inferred. Please remove language that implies causality, such as ‘effect/effectiveness’ throughout. Refer to associations instead.

**Response**: Done.

Abstract Methods and Findings:

* Please include the study design (cohort study), population and setting (patients attending primary care in England), and main outcome measures.

* In the last sentence of the Abstract Methods and Findings section, please describe the main limitations of the study's methodology.

Please update the Abstract heading ‘Interpretations’ to ‘Conclusions’.

Please remove the ‘Funding’ section from the Abstract.

**Response**: Done.

Thank you for providing an Author Summary. Under the question ‘What do these findings mean’, authors should reflect on the new knowledge generated by the research and the implications for practice, research, policy, or public health. Authors should also consider how the interpretation of the study’s findings may be affected by the study limitations. The current bullet points are more aligned with the question above (What did the researchers do and find?).

We request 2-3 single sentence bullet points per question in the Author Summary. The aim is to make your findings accessible to a wide audience that includes both scientists and non-scientists, so please just concentrate on getting across the key points. See https://journals.plos.org/plosmedicine/s/revising-your-manuscript for further guidance.

**Response**: Thank you, we have now updated that section to reflect your recommendations.

Please relocate citations such that they precede punctuation.

**Response**: Done.

Thank you for including a STROBE checklist. Please update the checklist to use Section headings and paragraph numbers (rather than page numbers and paragraph numbers) as the page numbers will change in the event of publication.

**Response**: Done.

Please ensure you cite your STROBE checklist and other items in the Supporting Information using the following guidance: https://journals.plos.org/plosmedicine/s/supporting-information

**Response**: We have added the prefixes in the supplementary file names – the supplementary file names are provided a the end of the document, as previously requested.

Please provide the unadjusted comparisons as well as the adjusted comparisons in Table 2 and include covariates that were adjusted for in the legend.

**Response**: Thank you for the suggestion, but we are not sure what the univariable analyses will add to the paper – the paper is already complicated, and we are reluctant to add another set of estimates that are of very little use and do not inform the modelling in any way. In terms of the legend, we assume this relates to Figure 2, and we have added another footnote to clarify the model from which the graph stems.

Please ensure the flags and footnotes are correct in Figure 1 (the symbol ^ is used but does not appear to have a corresponding footnote).

**Response**: Thank you, we have made this clearer and are now using a single footnote.

Comments from the reviewers:

Reviewer #2: Thanks for the revised manuscript and replies to the earlier questions.

Thanks for the codes included as the appendix - if there's a way to directly link to the codes on the website that would be handy.

**Response**: Thank you, there is, we have updated the paper with a direct permalink.

The reversed y-axis on a survival plot (cumulative incidence) is a practice recommended by Pocock and Altman (https://doi.org/10.1016/S0140-6736(02)08594-X), I believe here that it would allow for a more straightforward interpretation of the graph and allow you to directly label the y-axis (proportion converted to T2DM) rather than needing a title and displaying the function. This is not a crucial change and if you feel it should stay as is, then take this just as a recommendation.

**Response**: Thank you, we do not have strong feelings about this, like the reviewer – we have now updated as requested.

Similarly the standardised difference is recommended for matching studies, and makes it much easier for the reader to see that the difference between the groups is limited.

**Response**: Thank you, we have added it now.
