# Supplementary material for: Referral to the NHS Diabetes Prevention Programme and conversion from nondiabetic hyperglycaemia to type 2 diabetes mellitus in England: A matched cohort analysis
Source: PLoS Med. 2023 Feb 27;20(2):e1004177. doi: 10.1371/journal.pmed.1004177 (PMC9970065; doi:10.1371/journal.pmed.1004177)
Supplement: Attachment — Submitted filename: Response 4.docx [file pmed.1004177.s008.docx]

Comments from the Academic Editor:

In the introduction part, it's better to introduce life-style intervention first and then introduce the medication intervention part. Because this manuscript focuses on the participants of prediabetes, life-style intervention is still the first and most important way.

***Thank you for your comment. This has now been changed, the medication intervention part has been moved to lines 149-153***

Requests from the editors:

Lines 397-398: Please use round brackets (external) and square brackets (internal).

***Done, now lines 403-404.***

Line 163: Typo (full stop in place of comma).

***Thanks, edited (line169 current version)***

The ‘Contributorship statement’ and ‘Funding’ sections can be removed from the main text (both are captured as metadata via the submission form and will be published alongside the article).

**Done**
